# Structural Characterization and Hypoglycemic Effects of a Purified Polysaccharide From *Cinnamomum cassia*

**DOI:** 10.1002/fsn3.71999

**Published:** 2026-06-10

**Authors:** Liquan Zhou, Yimiao Zhou, Chen Ding, Lin Yang, Tianjin Ma, Xiao Liu, Zuowei Xiao

**Affiliations:** ^1^ Hunan Engineering and Technology Research Center for Health Products and Life Science, School of Pharmacy Hunan University of Chinese Medicine Changsha China; ^2^ Homologous Innovation Laboratory of Medicine and Food Hunan University of Chinese Medicine Changsha China; ^3^ Xiangyin Campus, Xiangxing College Hunan University of Chinese Medicine Yueyang China; ^4^ Guizhou Institute of Crop Germplasm Resources Guizhou Academy of Agricultural Sciences Guiyang China

**Keywords:** *Cinnamomum cassia* polysaccharide, functional food, hypoglycemic effect, oxidative stress, PI3K/AKT, structural characterization, type 2 diabetes

## Abstract

A purified polysaccharide, designated CCP1A, was extracted and isolated from *Cinnamomum cassia* bark using ultrasonic‐assisted composite enzymatic extraction followed by DEAE‐52 and Sephadex G‐100 column chromatography. CCP1A had a molecular weight of 8.161 kDa and was primarily composed of glucose and galacturonic acid, with minor amounts of rhamnose, arabinose, galactose, xylose, and mannose. Spectroscopic analyses (FT‐IR and ^1^H NMR) confirmed the coexistence of α‐ and β‐glycosidic linkages and a pyranose ring structure. The Congo red assay indicated a stable helical conformation, and transmission electron microscopy revealed dendritic, coiled aggregates. In a mouse model of type 2 diabetes induced by a high‐fat diet combined with low‐dose streptozotocin, oral administration of CCP1A (100, 200, and 300 mg/kg/day for 4 weeks) dose‐dependently reduced fasting blood glucose, improved glucose tolerance and lipid profiles (TG, TC, LDL‐C, and HDL‐C), and alleviated polydipsia, polyphagia, weight loss, as well as pancreatic pathological damage. Mechanistically, CCP1A suppressed intestinal α‐amylase and α‐glucosidase activities, promoted hepatic and muscle glycogen synthesis, upregulated the hepatic mRNA expression of GCK, GLUT2, PI3K, AKT, and Nrf2, downregulated PEPCK mRNA expression, increased serum levels of T‐SOD, GSH‐Px, CAT, and T‐AOC, and decreased MDA and GSP levels. These findings suggest that CCP1A exerts multi‐target hypoglycemic effects potentially involving inhibition of intestinal glucose absorption, modulation of insulin signaling‐related genes, promotion of glycogen synthesis, suppression of gluconeogenesis, and upregulation of Nrf2 mRNA. Hence, CCP1A represents a promising natural candidate for functional food development against type 2 diabetes.

## Graphical abstract

CCP1A, a polysaccharide purified from *Cinnamomum cassia* with a molecular weight of approximately 8.16 kDa and rich in glucose and galacturonic acid, exerts anti‐type 2 diabetes effects through a multi‐target synergistic mechanism. On one hand, it delays intestinal glucose absorption by inhibiting the activities of α‐amylase and α‐glucosidase in the small intestine. On the other hand, it upregulates the mRNA expression of PI3K, AKT, GCK, and GLUT2 while downregulating PEPCK expression in the liver, thereby promoting hepatic glycogen synthesis and inhibiting gluconeogenesis. Meanwhile, CCP1A upregulates Nrf2 mRNA expression to enhance the activities of antioxidant enzymes such as T‐SOD, GSH‐Px, and CAT, and reduces MDA levels, effectively alleviating oxidative stress under diabetic conditions. Through these synergistic mechanisms, CCP1A significantly lowers fasting blood glucose, improves glucose tolerance, regulates lipid profiles, alleviates pancreatic pathological damage and the classic symptoms of diabetes (polydipsia, polyphagia, polyuria, and weight loss), demonstrating its potential as a functional food against type 2 diabetes.

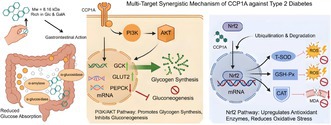

**Abbreviations**
AKT: Protein kinase B
ALT: Alanine aminotransferase
Ara: Arabinose
AST: Aspartate aminotransferase
AUCG: Area under the blood glucose curve
CAT: Catalase
FBG: Fasting blood glucose
Fru: Fructose
FT‐IR: Fourier transform infrared spectroscopy
Gal: Galactose
GalA: Galacturonic acid
GCK: Glucokinase
Glc: Glucose
GlcA: Glucuronic acid
GLUT2: Glucose transporter 2
GSH‐Px: Glutathione peroxidase
GSP: Glycated serum protein
HDL‐C: High‐density lipoprotein cholesterol
HE: Hematoxylin–eosin
LDL‐C: Low‐density lipoprotein cholesterol
LPS: Lipopolysaccharide
Man: Mannose
ManA: Mannuronic acid
MDA: Malondialdehyde
Nrf2: Nuclear factor E2‐related factor 2
OGTT: Oral glucose tolerance test
PEPCK: Phosphoenolpyruvate carboxykinase
PI3K: Phosphoinositide 3‐kinase
PMP: 1‐Phenyl‐3‐methyl‐5‐pyrazolone
qPCR: Quantitative polymerase chain reaction
Rha: Rhamnose
Rib: Ribose
ROS: Reactive oxygen species
SPF: Specific pathogen‐free
STZ: Streptozotocin
T2D: Type 2 diabetes
T‐AOC: Total antioxidant capacity
T‐CHO: Total cholesterol
TG: Triglyceride
T‐SOD: Total superoxide dismutase
UV: Ultraviolet
Xyl: Xylose

## 1 Introduction

Type 2 diabetes (T2D), characterized by insulin resistance and progressive β‐cell dysfunction, represents a global metabolic disorder (Shang et al. 2026). Chronic hyperglycemia‐driven oxidative stress functions as a critical mediator in diabetic complications development, directly impairing insulin signaling, triggering β‐cell apoptosis, and disrupting hepatic glucose and lipid metabolism (Zhou et al. 2025). Under hyperglycemic conditions, excessive reactive oxygen species (ROS) generation coupled with antioxidant defense system imbalance perpetuates metabolic dysfunction and oxidative damage.

First‐line therapeutics including metformin and α‐glucosidase inhibitors effectively regulate glycemic control; however, chronic administration frequently induces gastrointestinal adverse effects (Jayachandran et al. 2026). Consequently, investigation of natural products derived from medicinal‐edible plants possessing dual hypoglycemic and antioxidant properties has gained substantial research attention. Plant polysaccharides demonstrate distinctive advantages attributable to structural heterogeneity, favorable biocompatibility, and multi‐pathway regulatory potential (He et al. 2025). These compounds not only directly neutralize free radicals but also fundamentally enhance oxidative stress resistance through transcriptional pathway activation and endogenous antioxidant enzyme upregulation (Md Yusoff and Shafie 2026).

Cinnamon ( *Cinnamomum cassia* ), a traditional medicinal‐edible resource, contains water‐soluble polysaccharides (CCPs) regarded as principal bioactive constituents. Chen, Zhang, et al. (2025) developed an efficient ultrasonic‐assisted enzymatic extraction coupled with column chromatography purification protocol, yielding multiple structurally characterized acidic polysaccharide fractions. Although direct hypoglycemic evaluation was not conducted, differential suppression of lipopolysaccharide (LPS)‐induced macrophage inflammatory responses suggested potential anti‐diabetic efficacy via anti‐inflammatory mechanisms. Al‐Ajalein et al. (2023) isolated low‐molecular‐weight, low‐esterified pectin polysaccharides from cinnamon bark utilizing microwave‐assisted extraction with response surface optimization. Structural analysis confirmed low esterification degrees, with significant in vitro antioxidant capacity demonstrated. Zhang et al. (2022) further validated through animal experiments that cinnamon polysaccharides effectively lowered fasting blood glucose, ameliorated dyslipidemia, and enhanced hepatic and muscular glycogen deposition in diabetic mice.

Nevertheless, existing research predominantly emphasizes crude cinnamon polysaccharide in vitro activity screening, with inadequate systematic in vivo efficacy assessment. Zhang et al. (2024) utilized water‐extraction and alcohol‐precipitation methods to obtain cinnamon polysaccharides exhibiting potent in vitro α‐amylase and α‐glucosidase inhibition (IC_50_: 0.189 and 0.340 mg/mL, respectively) and substantial antioxidant activity, implying hypoglycemic potential through delayed carbohydrate digestion and absorption; yet comprehensive in vivo verification remained absent. More importantly, the dose‐dependent effects and systemic mechanisms by which highly purified, structurally well‐defined cinnamon polysaccharides improve overall metabolic phenotypes and organ pathological damage in T2D mouse models have not been fully elucidated.

To fill these research gaps, the present study employed ultrasound‐assisted complex enzyme extraction combined with DEAE‐52 and Sephadex G‐100 column chromatography to isolate and purify a polysaccharide (CCP1A) from cinnamon. Its purity, chemical composition, monosaccharide composition, molecular weight distribution, Congo red binding properties, ^1^H nuclear magnetic resonance (^1^H NMR) spectroscopy, and transmission electron microscopy (TEM) morphology were systematically characterized. Subsequently, utilizing a high‐fat diet combined with low‐dose streptozotocin (STZ)‐induced T2D murine model, CCP1A's in vivo therapeutic effects were comprehensively assessed across multiple dimensions: organismal level (glycemic and lipid profiles, dietary and water consumption, body mass dynamics), histopathological level (pancreatic tissue), and molecular level (intestinal digestive enzyme activity, hepatic glycogen metabolism, and associated gene expression). This multi‐faceted evaluation aims to establish a robust scientific foundation for CCP1A development and application.

## 2 Instruments and Materials

### 2.1 Instruments

Analytical balance (PX224ZH/E, Ohaus Instruments (Changzhou) Co. Ltd.), rotary evaporator (RE‐52C, Gongyi Zhongtian Instrument Technology Co. Ltd.), high‐speed refrigerated centrifuge (CenLee16R, Hunan Xiangli Scientific Instrument Co. Ltd.), ultrasonic cleaner (AK‐040SD, Shenzhen Yujie Cleaning Equipment Co. Ltd.), freeze dryer (A201‐10NA0279, Ningbo Scientz Biotechnology Co. Ltd.), UV spectrophotometer (UV1902PC, Shanghai Aoxi Scientific Instrument Co. Ltd.), Fourier Transform Infrared Spectroscopy (FT‐IR) spectrometer (Nicolet iS‐10, Thermo Fisher Scientific), HPLC system (UltiMate 3000, Thermo Fisher Scientific) coupled with refractive index (OPTILAB T‐rex, Wyatt Technology) and laser light scattering (DAWN HELEOS‐II, Wyatt Technology) detectors, nitrogen evaporator (Reacti‐thermo, Thermo Fisher Scientific), ion chromatography system (ICS 5000+, Thermo Fisher Scientific), multimode microplate reader (Varioskan LUX, Thermo Fisher Scientific), digital slide scanner (Pannoramic MIDI, 3Dhistech Ltd.), cryostat (CM3050S, Leica Microsystems), qPCR system (QuantStudio 7 Flex, Thermo Fisher Scientific), UV–Vis spectrophotometer (NanoDrop One, Thermo Fisher Scientific), glucometer (1015B250604, Sinocare Inc.), nuclear magnetic resonance spectrometer (Bruker DRX‐600, Bruker Corporation), and transmission electron microscope (Hitachi HT7800, Hitachi Ltd.) were utilized.

### 2.2 Materials

Sodium acetate anhydrous, sodium chloride, ethanol absolute, phosphoric acid, sodium tetraborate decahydrate, methanol, and isopropanol were procured from Sinopharm Chemical Reagent Co. Ltd. (Shanghai, China). Cellulase (50 U/mg), pectinase (50 U/mg), papain (800 U/mg), anthrone, DEAE cellulose DE‐52, Sephadex G‐100, standard monosaccharides including D‐fructose (Fru), D‐mannose (Man), L‐rhamnose (Rha), D‐xylose (Xyl), D‐glucuronic acid (GlcA), D‐galacturonic acid (GalA), D‐mannuronic acid (ManA), L‐guluronic acid sodium salt (GulA), L‐arabinose (Ara), D‐galactose (Gal), L‐fucose (Fuc), and D‐ribose (Rib), together with glycogen content assay kit, citrate buffer, glucose, metformin hydrochloride, glacial acetic acid, AB‐8 macroporous resin, physiological saline, methanol, absolute ethanol, phosphotungstic acid negative staining solution, and dialysis bag (2000 Da) were acquired from Shanghai Yuanye Bio‐Technology Co. Ltd. (Shanghai, China).

Trifluoroacetic acid (TFA), 1‐phenyl‐3‐methyl‐5‐pyrazolone (PMP), ethylenediaminetetraacetic acid (EDTA), sodium bicarbonate, and streptozotocin were sourced from Aladdin Reagent Co. Ltd. (Shanghai, China). Cinnamon medicinal materials were obtained from Hunan Zhenxing Traditional Chinese Medicine Co. Ltd. (Changsha, China). Chloroform, sulfuric acid, and hydrochloric acid were purchased from Hunan Huihong Reagent Co. Ltd. (Changsha, China). RNA isolater Total RNA Extraction Reagent was obtained from Nanjing Vazyme Biotech Co. Ltd. (Nanjing, China). 2× Universal SYBR Green Fast qPCR Mix and ABScript Neo RT Master Mix for qPCR were acquired from Wuhan ABclonal Biotechnology Co. Ltd. (Wuhan, China). Deuterium oxide (D_2_O) was purchased from Jinan Maina Technology Co. Ltd. (Jinan, China).

Assay kits for alanine aminotransferase (ALT), aspartate aminotransferase (AST), glycated serum protein (GSP), total cholesterol (T‐CHO), high‐density lipoprotein cholesterol (HDL‐C), low‐density lipoprotein cholesterol (LDL‐C), and triglyceride (TG) were supplied by Nanjing Jiancheng Bioengineering Institute (Nanjing, China). Colorimetric assay kits for total superoxide dismutase (T‐SOD), malondialdehyde (MDA), glutathione peroxidase (GSH‐Px), BCA protein concentration, catalase (CAT), total antioxidant capacity (T‐AOC), α‐glucosidase (α‐GC), and α‐amylase were procured from Wuhan Elabscience Biotechnology Co. Ltd. (Wuhan, China).

## 3 Methods

### 3.1 Preparation of CCP1A

Cinnamon bark was pulverized employing a swing mill and passed through a 60‐mesh standard sieve. The resulting powder was subjected to defatting and decolorization via reflux extraction with 95% ethanol at a liquid‐to‐solid ratio of 20 mL/g and 50°C for 4 h (Zhao et al. 2024). Following filtration, the residue was oven‐dried at 50°C to eliminate residual solvent.

The pretreated material underwent ultrasonic‐assisted composite enzymatic extraction. The enzymatic cocktail (cellulase:pectinase:papain = 8:1:1, w/w/w) was introduced at 2.92% loading, with pH adjusted to 4.5 using 0.05 mol/L acetic acid–sodium acetate buffer. Hydrolysis was performed at 50°C, a 20.5 mL/g liquid‐to‐solid ratio, 240 W ultrasonic power, for 91 min (Xiu et al. 2023). The extract was concentrated by rotary evaporation and then subjected to ethanol precipitation to a final concentration of 80% (v/v) at 4°C overnight. The resulting precipitate was collected by centrifugation (4500 r/min, 20 min) and lyophilized to obtain crude cinnamon polysaccharide (CCP).

The crude CCP was dissolved in distilled water to prepare a 10 mg/mL solution. Under ice‐bath cooling and magnetic stirring, an equal volume of 10% (w/v) trichloroacetic acid (TCA) solution was slowly added dropwise to the solution to achieve a final TCA concentration of 5% (w/v). After standing at 4°C for 5 h, the mixture was centrifuged at 5000 r/min for 20 min at 4°C, and the supernatant was carefully collected. The supernatant was transferred into a dialysis bag with a molecular weight cut‐off of 2000 Da and dialyzed against flowing distilled water for 48 h to remove small molecular impurities and TCA, during which the external dialysis water was replaced every 6 h. After dialysis, the solution inside the bag was concentrated and freeze‐dried to obtain a preliminarily deproteinized CCP sample.

Macroporous adsorption resin was used to further remove pigments and residual proteins from the above sample. The pre‐treated AB‐8 resin was wet‐packed into a column (2 × 60 cm) and equilibrated with distilled water. Then, 20 mL of the 10 mg/mL CCP solution was loaded onto the column at a flow rate of 2.0 mL/min. The column was eluted with distilled water followed by a 55% ethanol aqueous solution at a flow rate of 1.0 mL/min. The eluate was collected, concentrated, and freeze‐dried to obtain preliminarily purified CCP. After elution, the resin was regenerated with a 95% ethanol solution.

The preliminarily purified CCP was dissolved (20 mg/mL), filtered (0.45 μm), and applied to a DEAE‐52 cellulose column (3 × 50 cm) equilibrated with distilled water. Stepwise gradient elution was conducted using distilled water and NaCl solutions (0.1–0.5 mol/L, 0.1 mol/L increments) at 1.0 mL/min, collecting 6 mL/tube (11 tubes per gradient). Eluates were monitored by anthrone‐sulfuric acid method at 625 nm. Fractions corresponding to the 0.3 mol/L NaCl peak were pooled, concentrated, dialyzed, and lyophilized to obtain CCP1 (Liu, Wu, et al. 2025).

CCP1 was dissolved (20 mg/mL), filtered (0.22 μm), and chromatographed on a Sephadex G‐100 column (1.5 × 100 cm) pre‐equilibrated with distilled water. Isocratic elution with distilled water proceeded at 0.8 mL/min, collecting 6 mL/tube. The elution profile was monitored by the anthrone‐sulfuric acid method. Symmetrical peak fractions were combined, concentrated, desalted by dialysis, and lyophilized to yield purified polysaccharide CCP1A (Yang, Chu, et al. 2025).

### 3.2 Structural Characterization

#### 3.2.1 Polysaccharide Purity Determination

A glucose standard stock solution (0.1 mg/mL) was prepared by accurately weighing 10.0 mg of glucose, dissolving in distilled water, and diluting to 100 mL. Aliquots (0, 0.2, 0.4, 0.8, 1.2, 1.6, and 2.0 mL) were transferred to test tubes and adjusted to 2.0 mL with distilled water. Subsequently, 6.0 mL of 0.1% (w/v) anthrone‐sulfuric acid reagent was introduced. Following thorough mixing, the reaction was conducted in a boiling water bath for 10 min, then rapidly cooled in an ice bath to ambient temperature. Absorbance was recorded at 625 nm against a reagent blank (Zhang, Shi, et al. 2025). A standard curve was generated with glucose concentration (mg/mL) as the abscissa and absorbance as the ordinate. CCP1A samples were suitably diluted and assayed accordingly, with purity determined using Equation (1).

(1) $$\text{Polysaccharide Purity}\;\left(\%\right)=\frac{C\cdot V\cdot n}{m}\times 100\%$$

where C represents the sugar concentration (mg/mL) derived from the standard curve; V, the diluted sample volume (mL); n, the dilution factor; and m, the sample mass (mg).

#### 3.2.2 Protein Content Determination

Using bovine serum albumin (BSA) as the standard, prepare a stock solution at a concentration of 0.1 mg/mL to construct a standard curve. Aliquot 0, 0.1, 0.2, 0.3, 0.4, 0.5, and 0.6 mL of the stock solution into separate test tubes, and add distilled water to bring each volume to 1.0 mL. Add 5.0 mL of Coomassie Brilliant Blue G‐250 staining solution to each tube, mix, and let stand at room temperature for 10 min. Measure the absorbance at 595 nm. Plot the standard curve. Dilute CCP1A appropriately and carry out the same procedure. Calculate the protein content of CCP1A based on the standard curve.

#### 3.2.3 Uronic Acid Content Determination

Using D‐galacturonic acid as the standard, prepare a stock solution at a concentration of 0.1 mg/mL to construct a standard curve. Aliquot 0, 0.1, 0.2, 0.3, 0.4, 0.5, and 0.6 mL of the stock solution into separate test tubes, add distilled water to bring each volume to 1.0 mL, and pre‐cool in an ice bath for 5 min. Slowly add 6.0 mL of pre‐chilled sodium tetraborate‐sulfuric acid solution along the tube wall, mix thoroughly by shaking, heat in a boiling water bath for 5 min, and then cool in an ice bath. Add 100 μL of 0.15% (w/v) m‐hydroxybiphenyl solution, mix, and let stand at room temperature for 15 min. Measure the absorbance at 525 nm and plot the standard curve. Dilute CCP1A appropriately and determine under the same conditions. Calculate the uronic acid content based on the standard curve.

#### 3.2.4 Ultraviolet Scanning Spectroscopy

CCP1A was dissolved to prepare a 0.05 mg/mL solution and scanned spectrophotometrically across 200–600 nm. Absorption maxima at 260 nm (nucleic acids) and 280 nm (proteins) were examined to ascertain impurity elimination efficacy and polysaccharide purity.

#### 3.2.5 Monosaccharide Composition Analysis

CCP1A (5 mg) was accurately weighed into a hydrolysis tube, followed by the addition of 2 mL 2 mol/L TFA. The tube was sealed under a nitrogen atmosphere and subjected to hydrolysis at 121°C for 2 h. Upon cooling to ambient temperature, the hydrolysate was transferred to a centrifuge tube and evaporated to dryness under a nitrogen stream at 60°C. The residue was redissolved in 1 mL methanol and re‐evaporated; this dissolution‐evaporation cycle was repeated 2–3 times to ensure complete TFA elimination. Finally, the residue was dissolved in 1 mL ultrapure water and filtered through a 0.22 μm aqueous membrane for subsequent analysis.

Ion chromatography was performed using a CarboPac PA20 column coupled to a pulsed amperometric detector. The mobile phase comprised ultrapure water (A), 0.1 M NaOH (B), and 0.1 M NaOH containing 0.2 M NaAc (C). Gradient elution was programmed as follows: 0–26 min, linear gradient from 95:5:0 to 85:5:10 (A:B:C); 26–42 min, isocratic at 85:5:10; 42.1–52 min, linear gradient from 60:0:40 to 60:40:0; 52.1–60 min, return to 95:5:0 for equilibration. Flow rate was 0.5 mL/min, column temperature 30°C, and injection volume 5 μL.

Qualitative identification was achieved by comparing retention times with standard monosaccharides (Ara, GlcA, Rib, Man, GulA, Fuc, Rha, GalA, Glc, Xyl, ManA, Fru, Gal). Molar percentages were quantified by area normalization (Tian et al. 2025).

#### 3.2.6 Molecular Weight and Its Distribution Determination

Molecular weight and distribution were analyzed by gel permeation chromatography coupled with multi‐angle laser light scattering and refractive index detection (GPC‐MALLS‐RI). Samples were dissolved in 0.1 M NaNO_3_ containing 0.02% NaN_3_ (1 mg/mL), sonicated until complete dissolution, and filtered through a 0.45 μm membrane. Separation was performed on tandem gel columns (Ohpak SB‐805 HQ and SB‐803 HQ, 300 × 8 mm each) with 0.1 M NaNO_3_/0.02% NaN_3_ as mobile phase at 0.6 mL/min, 45°C column temperature, and 100 μL injection volume. Data were processed using ASTRA software to calculate number‐average (Mn), weight‐average (Mw), Z‐average (Mz) molecular weights, and polydispersity index (Mw/Mn) based on dn/dc values (Fu et al. 2025).

#### 3.2.7 FT‐IR Analysis

CCP1A (2 mg) was homogenized with 200 mg dried KBr in an agate mortar and compressed into 1 mm transparent pellets under 10 MPa. Spectra were recorded on a FT‐IR instrument at 4000 ~ 400 cm^−1^, 4 cm^−1^ resolution, with 32 scans (Medjdoub et al. 2025).

#### 3.2.8 Congo Red Assay

Prepare a 0.5 mg/mL CCP1A solution and a 50 μmol/L Congo Red solution. Mix 2 mL of CCP1A solution with 2 mL of Congo Red solution, then add appropriate volumes of 1 mol/L NaOH solution and distilled water to achieve final NaOH concentrations of 0, 0.1, 0.2, 0.3, 0.4, and 0.5 mol/L in the mixture. After reacting at room temperature in the dark for 20 min, scan the wavelength range of 400–600 nm using a UV–Vis spectrophotometer and record the change in the maximum absorption wavelength (λ_max_). A mixture containing Congo Red and the corresponding concentrations of NaOH but without CCP1A is used as a control.

#### 3.2.9 1H NMR Measurement

Accurately weigh 35 mg of CCP1A, dissolve it in 0.55 mL of deuterium oxide (D_2_O), and transfer the fully dissolved solution to an NMR tube. Acquire the ^1^H NMR spectrum using a Bruker DRX‐600 nuclear magnetic resonance spectrometer at 25°C. The sampling frequency is 600 MHz, and the residual HDO signal in D_2_O (δ 4.79 ppm) is used as an internal standard for chemical shift calibration (Gong et al. 2025).

#### 3.2.10 Transmission Electron Microscopy Measurement

Prepare a 0.1 mg/mL CCP1A solution and disperse it by ultrasonication for 10 min. Drop 10 μL of the sample solution onto a carbon‐coated copper grid, and let it stand at room temperature for 10 min to allow polysaccharide adsorption. Carefully remove excess liquid with filter paper, then add 10 μL of a 2% (w/v) phosphotungstic acid solution (pH 7.0) for negative staining for 1 min. Remove the staining solution and allow the grid to dry naturally at room temperature. Observe and photograph the microstructure of the sample using a Hitachi HT7800 transmission electron microscope at an accelerating voltage of 80 kV.

### 3.3 Hypoglycemic Activity

#### 3.3.1 Animal Feeding and Modeling

Specific pathogen‐free (SPF) male Kunming mice (4 weeks old) were acclimatized for 7 days and stratified by body weight into normal control (NC, *n* = 10) and modeling cohorts. NC animals received standard chow, whereas modeling animals were maintained on a high‐sucrose/high‐fat diet (66.5% basal feed, 10% lard, 20% sucrose, 2.5% cholesterol, 1% sodium cholate) for 4 weeks. Following 12‐h fasting with ad libitum water access for 5 consecutive days, diabetes was induced by intraperitoneal STZ injection (40 mg/kg/d in 0.1 mol/L citrate buffer, pH 4.5) (Wang et al. 2024). On day 8 post‐final injection, fasting blood glucose (FBG) was measured via tail vein sampling. Animals with FBG ≥ 16.7 mmol/L were deemed successfully diabetic.

Diabetic mice were randomized into 5 groups (*n* = 10) stratified by glycemic status and body mass: model control (MC), positive control (metformin 200 mg/kg/d plus acarbose 10 mg/kg/d), and CCP1A low‐ (100 mg/kg/d), medium‐ (200 mg/kg/d), and high‐dose (300 mg/kg/d) groups. The NC and MC groups received equivalent volumes of physiological saline (Xue et al. 2025). All treatments were administered by oral gavage at 09:00 daily for 4 weeks. Animals had ad libitum access to food and water throughout. Body mass, feed consumption, and water intake were monitored weekly.

#### 3.3.2 Physiological Parameter Measurements

Body mass was recorded weekly at consistent time points throughout the experimental period. Measurements were conducted on conscious animals with gentle handling to minimize stress‐induced artifacts. Weekly monitoring enabled dynamic evaluation of diabetes progression and therapeutic intervention effects on body mass (Zhang, Yang, et al. 2025).

Water and feed consumption were quantified gravimetrically. Pre‐weighed water bottles and chow were provided at fixed daily intervals; after 24 h, remaining bottles, feed, and spillage were collected and re‐weighed. Cage‐level consumption was calculated by mass difference and normalized to individual mice by dividing by cage occupancy to yield average daily water and feed intake (Yang, Wu, et al. 2025).

#### 3.3.3 Organ Coefficient Measurement

Following euthanasia, the heart, liver, spleen, and kidneys were promptly excised, rinsed with 0.9% saline to eliminate residual blood, blotted dry, and weighed. Organ coefficients were calculated according to Equation (2) (Ran et al. 2025):

(2) $$\begin{array}{c} \text{Organ Coefficient}\;\left(\%\right)=\frac{m1}{m2}\times 100\% \end{array}$$

where m1 represents the organ mass (g), and m2 represents the mouse body mass (g).

#### 3.3.4 FBG and Oral Glucose Tolerance Test (OGTT)

FBG was monitored at baseline (pre‐modeling) and on days 7, 14, 21, and 28 of treatment. Mice were fasted for 12 h with ad libitum water access prior to tail vein sampling (Qin et al. 2025).

Following 8 weeks of intervention, OGTT were performed. Animals were fasted for 12 h, then administered glucose (1 g/kg) by gavage. Blood was collected via tail puncture at 0, 30, 60, and 90 min post‐administration, with glucose concentrations determined using a glucometer (Song et al. 2025). Area under the glucose curve (AUCG, mmol·h/L) was calculated according to Equation (3):

(3) $$\begin{array}{c} \text{AUCG}=\frac{0.5A+B+C+0.5D}{2} \end{array}$$

where A, B, C, and D represent blood glucose at 0, 30, 60, and 90 min, respectively.

#### 3.3.5 Lipid Profile Assessment

Prior to terminal sampling, mice were fasted for 12 h. Body mass was recorded 1 h post‐final administration. Orbital venous blood was collected, centrifuged (4°C, 2500 r·min^−1^, 10 min), and serum was harvested. TG, T‐CHO, LDL‐C, and HDL‐C were quantified following manufacturer's protocols (Li et al. 2025).

#### 3.3.6 Pancreatic Tissue HE Staining and Observation

One piece of fresh pancreatic tissue from the same anatomical location was collected from each mouse in all groups and fixed in 4% paraformaldehyde solution for 24 h. After graded dehydration and xylene clearing, routine paraffin embedding, serial sectioning, and dewaxing to water were performed sequentially. Subsequently, the sections were stained using the hematoxylin–eosin (HE) method and mounted with neutral balsam. Histological structural changes were observed using a digital slide scanning analysis system, with a focus on evaluating the integrity of islet structure, the morphology of islet cells, and the degree of interstitial inflammatory infiltration (Matsathit et al. 2025).

#### 3.3.7 Detection of Liver Injury‐Related Markers

Serum ALT and AST activities were quantified colorimetrically to assess hepatocellular injury in diabetic mice (Omer et al. 2023).

#### 3.3.8 Determination of Serum Oxidative Stress Markers and Antioxidant Capacity

Serum GSH‐Px, T‐SOD, and CAT activities, alongside MDA, T‐AOC, and GSP levels, were quantified colorimetrically to evaluate oxidative stress status and short‐term glycemic control (Lin et al. 2025).

#### 3.3.9 Glycogen Detection and Digestive Enzyme Activity Analysis

Hepatic and muscular glycogen contents were quantified following manufacturer's protocols to assess CCP1A‐mediated regulation of glucose storage. Intestinal α‐amylase and α‐glucosidase activities were determined colorimetrically to elucidate CCP1A effects on intestinal glucose liberation (Lu et al. 2025).

#### 3.3.10 Quantitative Real‐Time PCR

Total RNA was extracted from 50 mg hepatic tissue using RNA isolator reagent, with purity and concentration assessed spectrophotometrically.

Reverse transcription was performed using ABScript Neo RT Master Mix for qPCR. The reaction system was prepared according to the manufacturer's instructions. The thermal cycling conditions were set as follows: 37°C for 2 min, 55°C for 15 min, 85°C for 5 min, and then held at 4°C. The obtained cDNA was immediately used for subsequent qPCR or stored at −20°C for short‐term preservation.

qPCR was performed on a real‐time fluorescence quantitative PCR system using 2× Universal SYBR Green Fast qPCR Mix. The amplification protocol was: pre‐denaturation at 95°C for 3 min; followed by 40 cycles of denaturation at 95°C for 5 s and annealing/extension at 60°C for 30 s. β‐actin was used as the internal reference gene. The relative mRNA expression levels of target genes, including glucokinase (GCK), glucose transporter 2 (GLUT2), phosphoenolpyruvate carboxykinase (PEPCK), phosphatidylinositol 3‐kinase (PI3K), protein kinase B (AKT), and nuclear factor erythroid 2‐related factor 2 (Nrf2), were calculated using the $2^{-\Delta \Delta C_{t}}$ method (Zhang, Chen, et al. 2025). Primer sequences used in this study are listed in Table 1.

**TABLE 1:** Primer Sequence Information.

Gene name	Forward primer	Reverse primer
β‐Actin	GGGAAATCGTGCGTGACATTA	TTGCCGATAGTGATGACCTGA
PEPCK	TTCCTGCCTCTCTCCACACC	CCCTGGATGACCTTGGCAGA
GCK	CAGTGAAATCCAGGCAAGGACAGG	TTCCAGGGGTAGCAGCAGAATAGG
GLUT2	GCACTCTGGCTGGTCAGCTATTC	AAGACAGTGAAAAGCCAAGGTTCCG
Nrf2	GCCTTCCTCTGCTGCCATTAGTC	TGCCTTCAGTGTGCTTCTGGTTG
AKT	TCAGGATGTGGATCAGCGAGAGTC	AGGCAGCGGATGATAAAGGTGTTG
PI3K	GGAATGTCGGGAGCAGCAACC	TCTACCACTACGGAGCAGGCATAG

#### 3.3.11 Statistical Analysis

Statistical analyses were conducted using SPSS 23.0. Continuous variables that followed a normal distribution were presented as mean ± standard deviation (SD). For body weight, food intake, water intake, and fasting blood glucose, which were measured longitudinally at multiple time points, two‐way repeated‐measures ANOVA was used to evaluate the main effects of group, time, and their interaction. For other indicators measured at a single time point, one‐way analysis of variance (ANOVA) was performed. For the two‐way repeated‐measures ANOVA, pairwise comparisons were carried out using Tukey's post hoc test. For the one‐way ANOVA, pairwise comparisons were performed using the least significant difference (LSD) *t*‐test. Statistical significance was denoted as *p < 0.05, **p < 0.01, and ***p < 0.001 versus the NC group; ^#^ *p <* 0.05, ^##^ *p <* 0.01, and ^###^ *p <* 0.001 versus the MC group. Graphs were generated using Origin 2021 and GraphPad Prism 10.1.2.

## 4 Results

### 4.1 Analysis of the Chromatographic Purification Process of CCP1A

**FIGURE 1:**
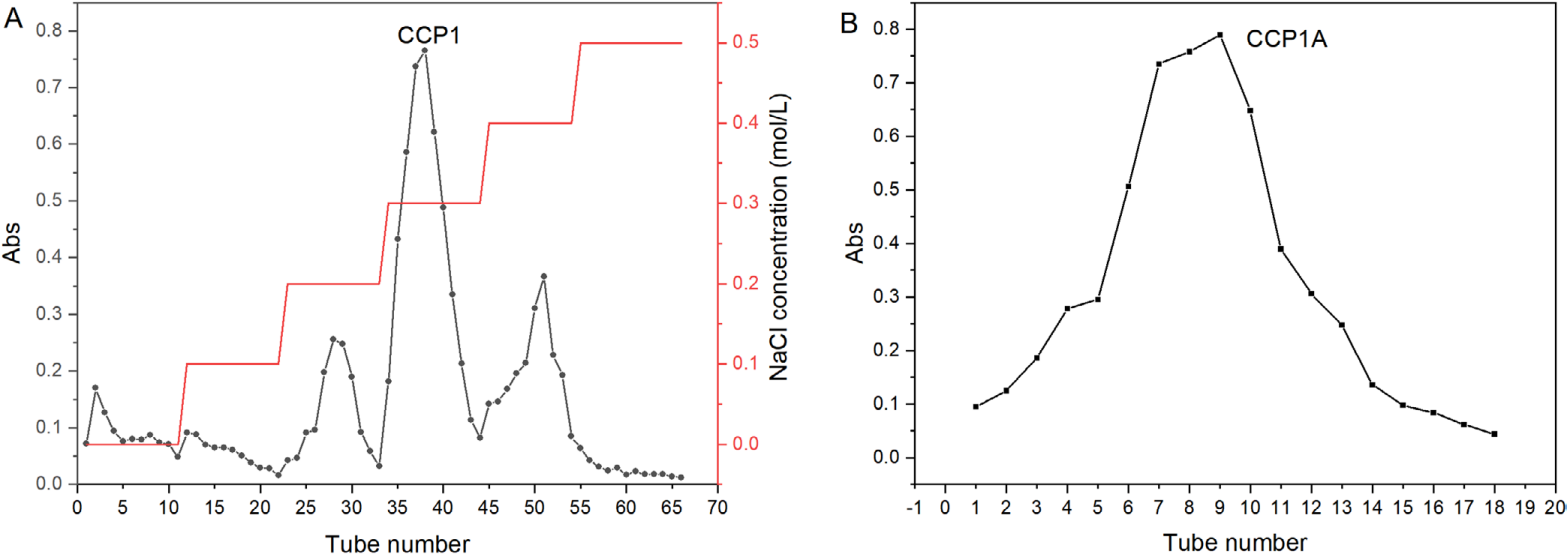
Chromatographic purification of CCP1 and CCP1A. (A) Elution profile on DEAE‐52; (B) Elution profile on Sephadex G‐100.

The preliminarily purified polysaccharide was subjected to DEAE‐52 ion exchange chromatography. Stepwise gradient elution with distilled water and NaCl (0.1–0.5 mol/L) yielded symmetrical, sharp peaks at distilled water, 0.2, 0.3, and 0.4 mol/L NaCl (Figure 1A). The 0.3 mol/L fraction (CCP1) exhibited the highest yield and collection volume, representing the predominant component of crude cinnamon polysaccharides. This elution profile suggests CCP1 is predominantly acidic.

CCP1 was subsequently chromatographed on Sephadex G‐100, producing a single symmetrical peak upon elution with distilled water (Figure 1B), confirming homogeneity. The main peak was pooled, concentrated, desalted by dialysis, and lyophilized to afford purified fraction CCP1A.

### 4.2 Structural Analysis

#### 4.2.1 Analysis of Polysaccharide Purity and Chemical Composition

**FIGURE 2:**
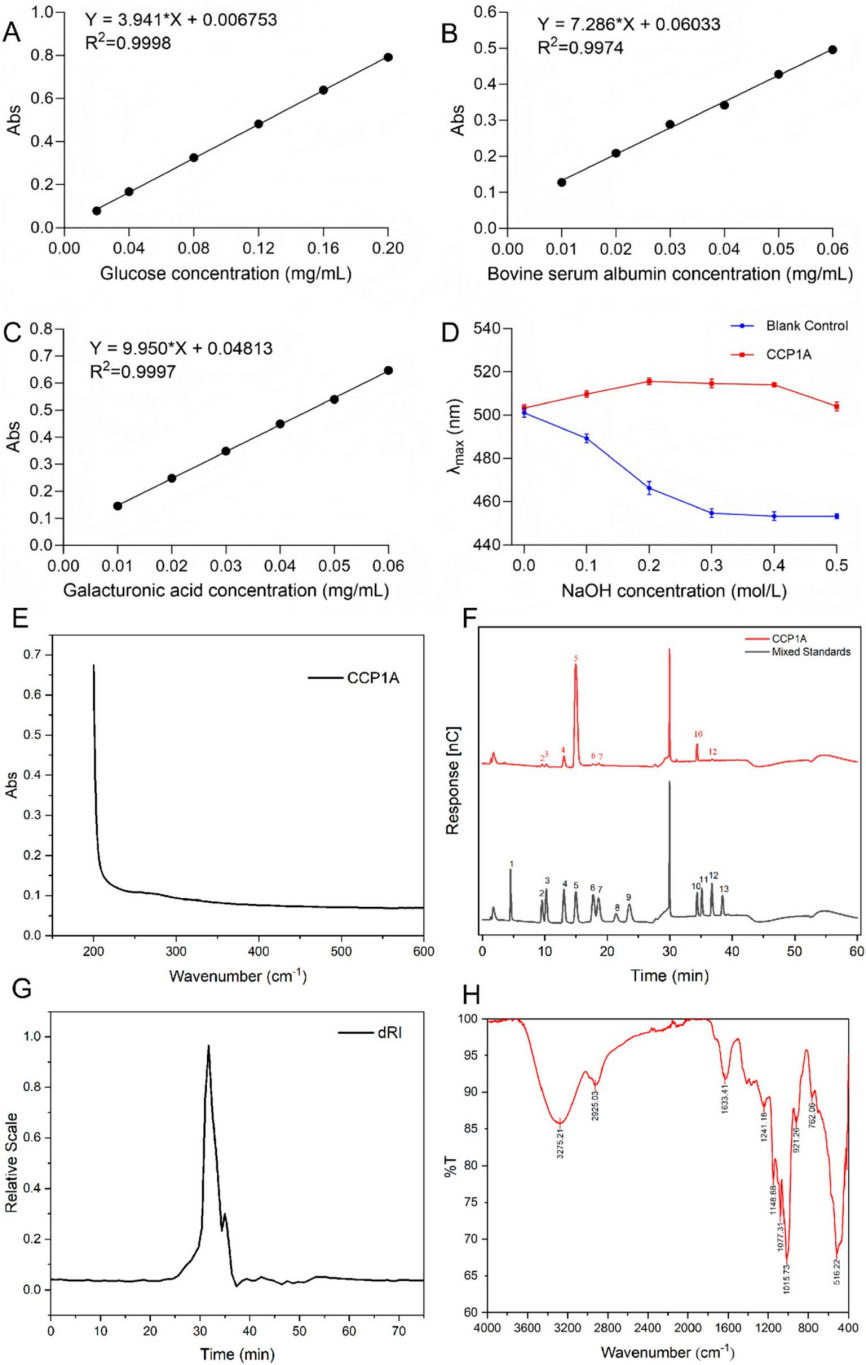
Structural characteristics of CCP1A. (A) Glucose standard curve, (B) bovine serum albumin standard curve, (C) galacturonic acid standard curve. (D) Congo red binding spectra of CCP1A and the blank control at different NaOH concentrations. (E) UV absorption spectrum. (F) Ion chromatography analysis. (G) GPC‐MALLS‐RI chromatogram. (H) FT‐IR spectrum. (F): 1‐Fuc, 2‐Ara, 3‐Rha, 4‐Gal, 5‐Glc, 6‐Xyl, 7‐Man, 8‐Fru, 9‐Rib, 10‐GalA, 11‐GulA, 12‐GlcA, 13‐ManA.

As shown in Figure 2A–C, the glucose standard curve is *y* = 3.941*x* + 0.006753 (*R* ^2^ = 0.9998), the bovine serum albumin standard curve is *y* = 7.286*x* + 0.06033 (*R* ^2^ = 0.9974), and the galacturonic acid standard curve is *y* = 9.950*x* + 0.04813 (*R* ^2^ = 0.9997), indicating good linearity. The purity (%), protein content (%), and uronic acid content (%) of CCP1A were 89.41 ± 0.95, 1.27 ± 0.11, and 23.46 ± 0.21, respectively.

#### 4.2.2 UV–Vis Spectroscopic Analysis

CCP1A exhibited negligible absorbance beyond 200 nm with no characteristic peaks at 280 nm (protein) or 260 nm (nucleic acid) (Figure 2E), confirming effective elimination of protein and nucleic acid impurities.

#### 4.2.3 Monosaccharide Composition

Ion chromatography analysis revealed that the molar ratio of monosaccharide composition in CCP1A was Rha:Ara:Gal:Glc:Xyl:Man:GalA:GlcA = 2.77:2.52:8.81:114.85:1.00:1.97:28.00:1.37 (Figure 2F). Glucose predominated (approximately 71.7%), followed by galacturonic acid (approximately 17.5%), indicating CCP1A is a glucose‐ and uronic acid‐rich heteropolysaccharide. The elevated uronic acid content likely contributes to its ion chelating and radical scavenging activities (Liu, Li, et al. 2025).

Structural parameters calculated via Formulas ((4), (5), (6)) yielded R1 ≈1.99, R2 ≈0.10, and R3 ≈4.09. R1 > 1 indicates a significant proportion of linear homogalacturonan (HG) domains, suggesting that the polysaccharide backbone contains many extended, unbranched regions. The relatively low R2 value indicates a small proportion of rhamnogalacturonan I (RG‐I) main chain (Zhang, Wei, et al. 2025). Moreover, R3 > 4 reflects a high degree of branching in the RG‐I region, with side chains composed mainly of arabinose and galactose.

Numerous studies have demonstrated that the monosaccharide composition of cinnamon polysaccharides is primarily composed of glucose, galactose, galacturonic acid, and other monosaccharides (Al‐Ajalein et al. 2023; Zhang et al. 2024). The dominant glucose residues in cinnamon polysaccharides can form a glucan backbone through →4)‐Glc‐(1 → and →6)‐Glc‐(1 → linkages (Chen et al. 2026; Dong et al. 2026). This glucan chain structure helps promote hepatic glycogen synthesis, upregulate GCK/GLUT2, downregulate PEPCK, and directly ameliorate hepatic glucose metabolic disorders. The uronic acid content is associated with antioxidant and immunomodulatory activities, and a high galacturonic acid content may confer anionic characteristics to CCP1A (Chen, Zhang, et al. 2025). Minor amounts of mannose, arabinose, and galactose exist as branch structures, which contribute to improving the water solubility and bioavailability of the polysaccharide and enhance its binding capacity to target proteins.

(4) $$\begin{array}{c} R1=\frac{\text{GalA}}{\mathrm{Rha}+\mathrm{Ara}+\mathrm{Gal}} \end{array}$$

(5) $$\begin{array}{c} R2=\frac{\mathrm{Rha}}{\text{GalA}} \end{array}$$

(6) $$\begin{array}{c} R3=\frac{\mathrm{Ara}+\mathrm{Gal}}{\mathrm{Rha}} \end{array}$$

#### 4.2.4 Molecular Weight and Its Distribution

As shown in Figure 2G, a distinct elution peak appeared at a retention time of approximately 30 min; however, the peak shape exhibited a gradual decline on the higher retention time side, indicating that in addition to the main component, the sample also contained a certain proportion of low‐molecular‐weight fragments, resulting in peak tailing (Wen, Zhou, et al. 2025).

The peak molecular weight (Mp) was 4.486 kDa, representing the molecular weight of the most abundant component. The number‐average molecular weight (Mn) was 6.198 kDa, which was higher than Mp, suggesting that the sample simultaneously contained a considerable proportion of high‐molecular‐weight components, thereby elevating the overall number‐average value. The weight‐average molecular weight (Mw = 8.161 kDa) and the z‐average molecular weight (Mz = 15.137 kDa) increased successively and significantly, indicating that the molecular weight distribution extends toward the higher molecular weight region. The polydispersity index (Mw/Mn) was 1.317, falling within the range of 1–1.5, which indicates that CCP1A exhibits a moderately broad molecular weight distribution—neither a monodisperse system nor an extremely broad distribution. Combined with the chromatogram and molecular weight data, it can be inferred that the polysaccharide sample has a core molecular weight range of 4–6 kDa, accompanied by a continuous distribution that gradually diminishes toward higher molecular weights (extending to approximately 15 kDa).

#### 4.2.5 FT‐IR Analysis

As shown in Figure 2H, the strong, broad absorption peak at 3275 cm^−1^ originates from the stretching vibrations of the abundant hydroxyl groups (–OH) in CCP1A molecules. The medium‐intensity peak at 2925 cm^−1^ is assigned to the stretching vibrations of saturated C–H bonds, presumably arising mainly from the methylene groups of pyranose rings such as glucose and galactose, as well as a minor contribution from the methyl groups of rhamnose. In the region of 1241–1015 cm^−1^, a continuous group of strong absorptions is observed, which constitutes the fingerprint region of CCP1A. Among these, the shoulder peak near 1241 cm^−1^ may include contributions from the C–O stretching of uronic acids, consistent with the galacturonic acid content, while the multiple absorptions in the range of 1150–1030 cm^−1^ correspond to the stretching vibrations of glycosidic bonds (C–O–C) and the C–O–H groups of sugar rings, reflecting the complex glycosidic linkage patterns of CCP1A as a polysaccharide (Gong et al. 2025). The characteristic peak at 921 cm^−1^ is unequivocally assigned to the skeletal vibration of pyranose rings, confirming that the sugar units exist in the pyranose form. In the low‐wavenumber region, the weak absorptions at 762 and 516 cm^−1^ originate from the skeletal bending vibrations of the sugar rings; the band at 762 cm^−1^ is sensitive to the α‐configuration, and the band at 516 cm^−1^ reflects ring‐folding vibrations. Together, these two bands indicate the structural complexity arising from the coexistence of α‐ and β‐glycosidic linkages in CCP1A.

#### 4.2.6 Congo Red Assay Analysis

Under non‐alkaline conditions, the λmax of the CCP1A‐Congo Red complex exhibited a significant red shift compared to the blank control group, preliminarily confirming the formation of the complex and suggesting that CCP1A may possess a helical structure (Figure 2D). As the NaOH concentration increased, the λmax of the blank control group showed a rapid and substantial blue shift, revealing the instability of Congo Red itself under alkaline conditions. In contrast, although the λmax of the CCP1A complex system decreased slightly, the magnitude of the change was minimal, and it remained at a high level. This pronounced difference in stability demonstrates that the helical structure of CCP1A can tightly bind Congo Red and effectively shield the chromophore of the dye from alkaline damage.

#### 4.2.7 1H NMR Analysis

**FIGURE 3:**
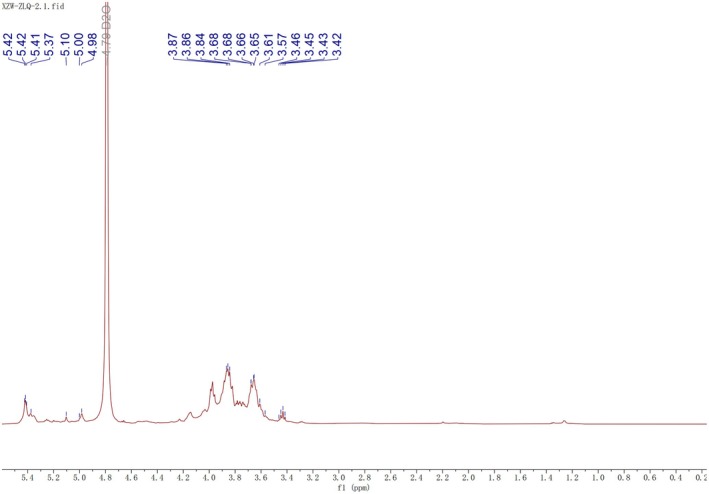
^1^H NMR spectrum of CCP1A.

In the low‐field anomeric hydrogen region (δH 4.8–5.5 ppm), several characteristic signals were observed (Figure 3). Among them, a relatively strong signal near δH ~5.42 ppm is typically assigned to the anomeric hydrogen (H1) of α–configurational glycosidic bonds. Its relatively high intensity is consistent with the fact that glucose accounts for a very high proportion of the monosaccharide composition, suggesting the possible presence of a glucan backbone (e.g., α–1,4– or α–1,6–linked) that is mainly α–linked. Meanwhile, signals observed around δH ~5.10 ppm and below 5.0 ppm may correspond to the anomeric hydrogens of other α–configurational sugar residues (e.g., α–linked galacturonic acid) and β–configurational glycosidic bonds (e.g., β–linked galactose, xylose, etc.), respectively (Chen et al. 2023). Due to the subtle and complex differences in the proton chemical environments of different sugar residues and linkage positions in CCP1A, extensive signal overlap occurs, forming a typical “fingerprint region” of complex polysaccharides. In the high‐field region (δH 3.4–4.8 ppm), the spectrum displays highly dense and severely overlapping broad peak clusters. The signals in this region originate from the non–anomeric protons at the C2–C6 positions of all sugar residues.

#### 4.2.8 Transmission Electron Microscopy Analysis

**FIGURE 4:**
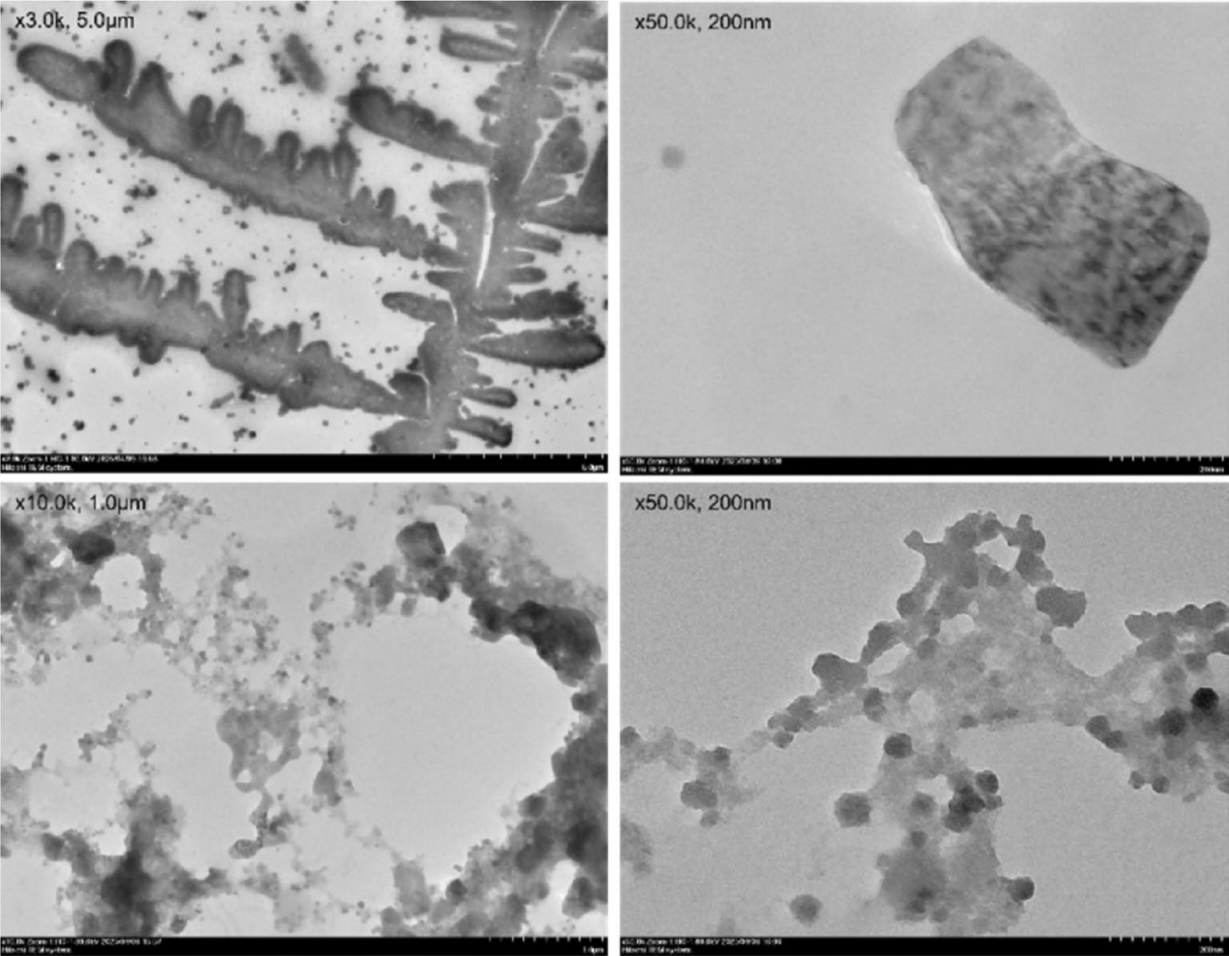
Multi‐scale TEM morphology of CCP1A.

In low‐magnification fields, CCP1A appeared as dendritic, irregular, and dispersed micron‐sized aggregates (Figure 4). The internal structure of the aggregates was heterogeneous, with loose and dense regions interweaving, exhibiting a fibrous or distinctly coiled chain‐like morphology. The continuous bending and coiling tendency of the chain segments corroborate the helical conformation inferred from the previous Congo Red assay.

### 4.3 Hypoglycemic Activity Analysis

#### 4.3.1 Body Weight Change Analysis

The body weight of mice in the NC group increased steadily throughout the entire observation period, reflecting the normal growth pattern under physiological conditions (Table 2). In the MC group, body weight was significantly lower than that of the NC group after model induction at week 5 (*p <* 0.001) and exhibited a continuous decline, suggesting that the T2D model successfully induced metabolic disorders and suppressed body weight gain in the mice. The body weight of the PC group became significantly higher than that of the MC group from week 7 onward (*p <* 0.001), indicating that the positive drug effectively alleviated the body weight loss in the diabetic mice. In the CL, CM, and CH groups, body weight remained significantly lower than that of the NC group after week 6 (*p <* 0.01) but was significantly higher than that of the MC group from week 7 onward (*p <* 0.05), demonstrating that CCP1A exerted a clear ameliorative effect on the body weight decline in T2D mice. Among these, the CH group exhibited the most pronounced body weight recovery, with effects comparable to those of the PC group, suggesting that the high‐dose cinnamon polysaccharide offers a more prominent body weight‐preserving effect.

**TABLE 2:** Body weight changes in mice from different groups (Weeks 4–9).

Group	Body weight (g)				
	Wk 4	Wk 6	Wk 7	Wk 8	Wk 9
NC	49.46 ± 1.77	51.28 ± 2.67	52.39 ± 2.48	53.19 ± 2.69	53.99 ± 2.97
MC	47.31 ± 1.56	44.82 ± 2.05***	43.27 ± 1.87***	44.12 ± 1.79***	44.97 ± 1.69***
PC	47.59 ± 1.83	45.32 ± 1.85***	46.62 ± 1.79***^,^^##^	47.22 ± 1.82***^,^^##^	47.82 ± 1.87***^,^^#^
CL	48.78 ± 2.02	44.82 ± 2.18***	47.02 ± 2.18***^,^^###^	47.6 ± 2.21***	48.18 ± 2.25***^,^^##^
CM	47.17 ± 2.32	45.18 ± 2.24***	45.89 ± 2.23***^,^^#^	46.16 ± 2.09***^,^^##^	46.44 ± 1.99***
CH	48.8 ± 1.54	45.93 ± 1.86***	47.93 ± 1.86***^,^^###^	48.16 ± 1.85***^,^^###^	48.39 ± 1.85***^,^^##^

*Note:* All data are shown as mean ± SD (*n* = 10). ***p < 0.001 versus the NC group, and ^#^ *p <* 0.05, ^##^ *p <* 0.01, ^###^ *p <* 0.001 versus the MC group.

#### 4.3.2 Water Intake Analysis

The water intake of mice in the NC group remained stable at 9–10 g throughout the entire observation period, reflecting normal drinking behavior under physiological conditions (Table 3). In the MC group, water intake was significantly higher than that of the NC group after model induction at week 5 (*p <* 0.01) and exhibited a continuous upward trend, reaching 12.36 g at week 9, suggesting that the T2D model successfully induced polydipsia in the mice, consistent with a typical pathological manifestation of diabetes. Water intake in the PC group was significantly higher than that in the NC group at weeks 6 and 7 (*p <* 0.01), but became significantly lower than that in the MC group from week 8 onward (*p <* 0.01), indicating that the positive drug effectively alleviated the polydipsia symptoms in the diabetic mice. In the CL, CM, and CH groups, water intake remained significantly higher than that in the NC group after week 6 (*p <* 0.05); however, from week 7 or 8 onward, water intake in the CM and CH groups was significantly lower than that in the MC group (*p <* 0.05), and the CL group also showed a trend toward improvement. Notably, water intake in the CM group approached the NC group level at weeks 8 and 9, demonstrating that CCP1A exerted a clear ameliorative effect on the polydipsia symptoms in T2D mice, with more pronounced effects observed in the medium‐ and high‐dose intervention groups.

**TABLE 3:** Daily water intake in mice from different groups (Weeks 4–9).

Group	Water intake (g)				
	Wk 4	Wk 6	Wk 7	Wk 8	Wk 9
NC	10.20 ± 1.15	10.00 ± 0.68	9.46 ± 0.63	9.83 ± 0.45	9.15 ± 0.5
MC	10.22 ± 0.63	11.99 ± 1.24**	12.16 ± 0.76***	12.53 ± 0.63***	12.36 ± 1.34***
PC	9.93 ± 1.04	12.10 ± 0.51**	11.27 ± 1.22**	10.78 ± 1.25^##^	10.82 ± 1.15*^#^
CL	10.71 ± 1.05	11.73 ± 1.08*	11.83 ± 0.95***	11.33 ± 1.19*	11.03 ± 1.23**
CM	10.75 ± 0.58	11.89 ± 1.13**	10.82 ± 1.09	10.40 ± 1.36^###^	10.17 ± 1.62^###^
CH	10.47 ± 0.98	11.85 ± 1.04**	11.70 ± 1.37***	11.11 ± 0.99^#^	10.58 ± 1.19*^##^

*Note:* All data are shown as mean ± SD (*n* = 10). *p < 0.05, **p < 0.01, ***p < 0.001 versus the NC group, and ^#^ *p <* 0.05, ^##^ *p <* 0.01, ^###^ *p <* 0.001 versus the MC group.

#### 4.3.3 Food Intake Analysis

As shown in Table 4, the food intake of mice in the NC group remained stable throughout the entire observation period, reflecting normal feeding behavior under physiological conditions. After model induction at week 6, the food intake in the MC group was significantly higher than that in the NC group (*p <* 0.001) and exhibited a continuous upward trend, reaching 9.88 g at week 9, suggesting that the T2D model successfully induced polyphagia in the mice. The food intake in the PC group remained significantly higher than that in the NC group at weeks 6–7 (*p <* 0.05), but became significantly lower than that in the MC group from week 8 onward (*p <* 0.001) and fell back to a level close to that of the NC group, indicating that the positive drug effectively alleviated polyphagia in the diabetic mice. In the CL group, food intake was significantly higher than that in the NC group at weeks 6–7 (*p <* 0.05) and was only slightly lower than that in the MC group at week 9 (*p <* 0.05), demonstrating a relatively limited ameliorative effect. In the CM group, food intake was significantly higher than that in the NC group at weeks 6–8 (*p <* 0.05), but was significantly lower than that in the MC group at week 9 (*p <* 0.001), with food intake approaching the NC group level. In the CH group, food intake was significantly lower than that in the MC group starting from week 7 (*p <* 0.01), and already approached the NC group level at weeks 8–9, indicating that CCP1A exerted an ameliorative effect on polyphagia in T2D mice, with more pronounced effects observed in the medium‐ and high‐dose intervention groups (Ullah et al. 2025).

**TABLE 4:** Daily food intake in mice from different groups (Weeks 4–9).

Group	Food intake (g)				
	Wk 4	Wk 6	Wk 7	Wk 8	Wk 9
NC	7.82 ± 0.16	7.79 ± 0.55	7.82 ± 0.41	7.86 ± 0.59	7.96 ± 0.35
MC	8.27 ± 0.45	9.62 ± 1.06***	9.77 ± 0.90***	9.67 ± 1.18***	9.88 ± 0.99***
PC	7.70 ± 0.79	8.79 ± 0.79*	8.82 ± 0.69*	7.29 ± 1.13^###^	7.73 ± 0.75^###^
CL	8.32 ± 0.69	9.16 ± 0.63**	9.00 ± 0.76**	8.84 ± 0.70*	8.83 ± 0.52^#^
CM	7.83 ± 0.56	9.05 ± 0.36**	8.98 ± 0.66*	8.87 ± 0.67*	8.16 ± 0.59^###^
CH	7.88 ± 0.58	8.80 ± 0.66*	8.41 ± 0.66^##^	8.23 ± 0.89^###^	8.28 ± 0.45^###^

*Note:* All data are shown as mean ± SD (*n* = 10). *p < 0.05, **p < 0.01, ***p < 0.001 versus the NC group, and ^#^ *p <* 0.05, ^##^ *p <* 0.01, ^###^ *p <* 0.001 versus the MC group.

#### 4.3.4 Organ Coefficients

As shown in Table 5, the organ indices of the NC group remained at stable physiological baseline levels. In the MC group, the liver index was significantly higher than that of the NC group (*p <* 0.001); the spleen index was also slightly elevated, the heart index showed a mild upward trend, and the kidney index exhibited no significant difference compared with the NC group, suggesting that the diabetic model successfully induced pathological changes in the organs, especially the liver, of the mice. After intervention in the PC group, the liver index was significantly lower than that of the MC group (*p <* 0.05), but the spleen index was significantly higher than that of the NC group (*p <* 0.05), while the heart and kidney indices showed no significant differences from those of the MC group (Wen, Zhang, et al. 2025).

**TABLE 5:** Organ coefficients in mice from different groups.

Group	Organ coefficients (%)			
	Heart	Hepatic	Spleen	Kidney
NC	0.46 ± 0.03	4.08 ± 0.4	0.23 ± 0.05	1.39 ± 0.14
MC	0.48 ± 0.04	5.35 ± 0.59***	0.29 ± 0.04	1.42 ± 0.13
PC	0.47 ± 0.03	4.72 ± 0.21*^,^^#^	0.31 ± 0.03*	1.35 ± 0.14
CL	0.45 ± 0.03	4.76 ± 0.3*	0.32 ± 0.04**	1.38 ± 0.1
CM	0.45 ± 0.03	4.98 ± 0.43**	0.28 ± 0.05	1.29 ± 0.09
CH	0.42 ± 0.04^#^	4.9 ± 0.23**	0.28 ± 0.05	1.27 ± 0.09

*Note:* All data are shown as mean ± SD (*n* = 10). *p < 0.05, **p < 0.01, ***p < 0.001 versus the NC group, and ^#^ *p <* 0.05 versus the MC group.

The regulatory effects of CCP1A at different doses on organ indices exhibited organ‐ and dose‐specificity. For the heart index, the CH group was significantly lower than the MC group (*p <* 0.05), whereas the CL and CM groups were similar to the MC group. For the liver index, all CCP1A dose groups significantly reduced the elevated liver index observed in the MC group, and the liver index of the CL group was the closest to that of the NC group. For the spleen index, the CL group was significantly higher than the NC group (*p <* 0.01), while the CM and CH groups were comparable to the MC group. For the kidney index, the CM and CH groups exhibited a decreasing trend compared with the MC group, whereas the CL group was close to the MC group. These results suggest that CCP1A regulates organ indices in diabetic mice in an organ‐ and dose‐dependent manner and can ameliorate diabetes‐related organ pathological changes to a certain extent.

#### 4.3.5 FBG and OGTT Analysis

The FBG in the NC group remained at a stable, normal level throughout the entire observation period. Compared with the NC group, the FBG in the MC group was elevated from week 5 onward and remained persistently at a high level (*p <* 0.001), indicating the successful induction of the T2D model (Table 6). The FBG in the PC group began to decline markedly from week 6 and gradually approached the NC group level, with significant differences from the MC group observed from week 6 to week 9 (*p <* 0.001), demonstrating that the positive drug effectively reduced blood glucose in the diabetic mice. The FBG in the CL, CM, and CH groups all showed a downward trend from week 6 onward, and each treatment group exhibited significant improvement compared with the MC group from week 6 (*p <* 0.05). Among them, the CH group displayed the greatest and most stable reduction in blood glucose, with FBG approaching the NC group level after week 7, indicating that CCP1A ameliorates FBG in T2D mice, with the most prominent effect observed in the high‐dose intervention group.

**TABLE 6:** Fasting blood glucose (FBG) levels in mice from different groups (Weeks 5–9).

Group	FBG (mmol/L)				
	Wk 5	Wk 6	Wk 7	Wk 8	Wk 9
NC	6.73 ± 1.87	7.08 ± 1.25	6.93 ± 1.08	7.62 ± 1.38	7.47 ± 1.40
MC	20.67 ± 1.87***	20.82 ± 3.73***	21.65 ± 3.26***	20.17 ± 2.69***	21.68 ± 1.55***
PC	20.29 ± 1.83***	14.22 ± 1.88***^,^^###^	12.68 ± 2.57***^,^^###^	12.63 ± 2.56**^,^^###^	9.98 ± 1.41^###^
CL	20.05 ± 1.07***	16.43 ± 2.73***^,^^##^	13.12 ± 3.7***^,^^###^	11.05 ± 3.94^###^	10.43 ± 3.88^###^
CM	20.69 ± 1.73***	17.12 ± 1.95***^,^^#^	12.78 ± 2.29***^,^^###^	11.20 ± 2.69*^,^^###^	10.88 ± 2.85^###^
CH	20.24 ± 1.70***	17.02 ± 1.35***^,^^#^	9.55 ± 1.98^###^	9.15 ± 1.50^###^	8.38 ± 2.19^###^

*Note:* All data are shown as mean ± SD (*n* = 10). *p < 0.05, **p < 0.01, ***p < 0.001 versus the NC group, and ^#^ *p <* 0.05, ^##^ *p <* 0.01, ^###^ *p <* 0.001 versus the MC group.

**FIGURE 5:**
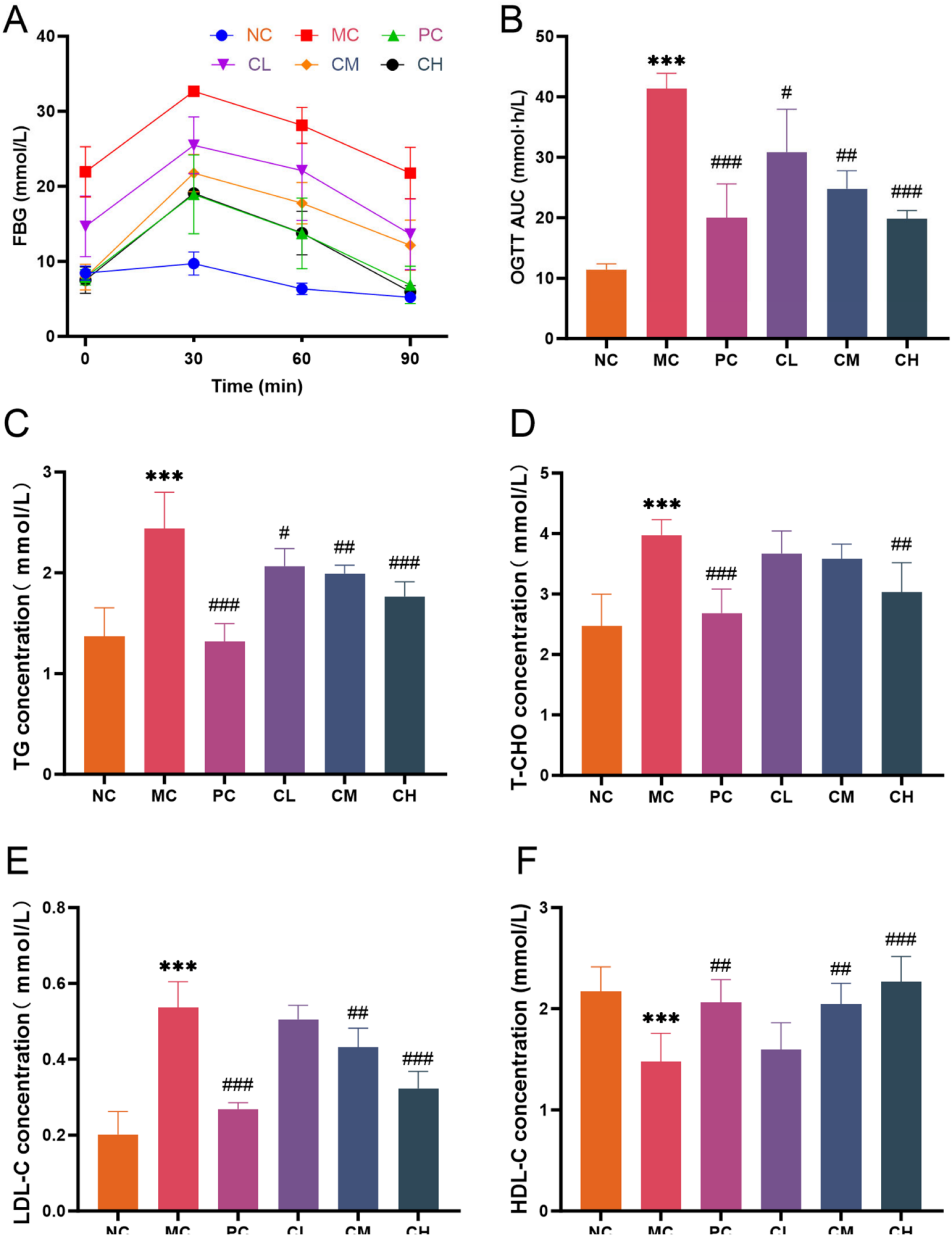
Oral glucose tolerance test (OGTT) curves (A), area under the glucose curve (AUCG) values (B), and serum lipid profiles—TG (C), T‐CHO (D), LDL‐C (E), HDL‐C (F)—in mice from different groups. All data are shown as mean ± SD (*n* = 10). ***p < 0.001 versus the NC group, and ^#^ *p <* 0.05, ^##^ *p <* 0.01, ^###^ *p <* 0.001 versus the MC group.

Based on the dynamic glucose tolerance curves in Figure 5A and the area under the glucose curve (AUCG) results in Figure 5B, the NC group exhibited mild blood glucose fluctuations after the glucose load and consistently remained at a low level, reflecting normal glucose metabolic capability. In the MC group, blood glucose was already elevated at the initial time point of glucose administration, rose rapidly to a peak after the glucose load, and subsequently declined slowly; its AUCG was significantly higher than that of the NC group (*p <* 0.001), indicating severely impaired glucose tolerance in the T2D model mice (Wang et al. 2025). The blood glucose fluctuation amplitudes in the PC, CL, CM, and CH groups were all significantly smaller than those in the MC group, with the CH group showing a glucose curve closest to that of the NC group, characterized by lower blood glucose levels at each time point and faster recovery. In terms of AUCG values, the PC, CL, CM, and CH groups all exhibited significantly lower AUCG than the MC group (*p <* 0.05), and the CH group had the lowest AUCG, approaching the NC group level, suggesting the most favorable recovery of glucose tolerance. The ameliorative effects of the low‐ and medium‐dose groups were relatively weaker than that of the high‐dose group.

#### 4.3.6 Lipid Profile Analysis

Based on the serum lipid profiles including HDL‐C, LDL‐C, total cholesterol (TC), and triglycerides (TG), the lipid parameters of mice in the NC group remained at stable physiological levels (Figure 5C–F). In the MC group, the levels of TG, TC, and LDL‐C were significantly higher than those in the NC group (*p <* 0.001), while the HDL‐C level was significantly lower (*p <* 0.001), indicating that the high‐fat diet successfully induced dyslipidemia in the mice. Compared with the MC group, the PC group exhibited significantly reduced TG, TC, and LDL‐C levels (*p <* 0.001) and a significantly elevated HDL‐C level (*p <* 0.01), demonstrating that the positive drug effectively reversed the high‐fat diet‐induced dyslipidemia. The CL, CM, and CH groups all showed varying degrees of reduction in serum TG, TC, and LDL‐C levels and an increase in HDL‐C level compared with the MC group, in a clear dose‐dependent manner. Among them, the CH group displayed the most pronounced regulatory effect, with some parameters approaching the levels of the PC group, suggesting that CCP1A can ameliorate hyperlipidemia by regulating lipid metabolism (Xu et al. 2025).

#### 4.3.7 Histopathological Findings

**FIGURE 6:**
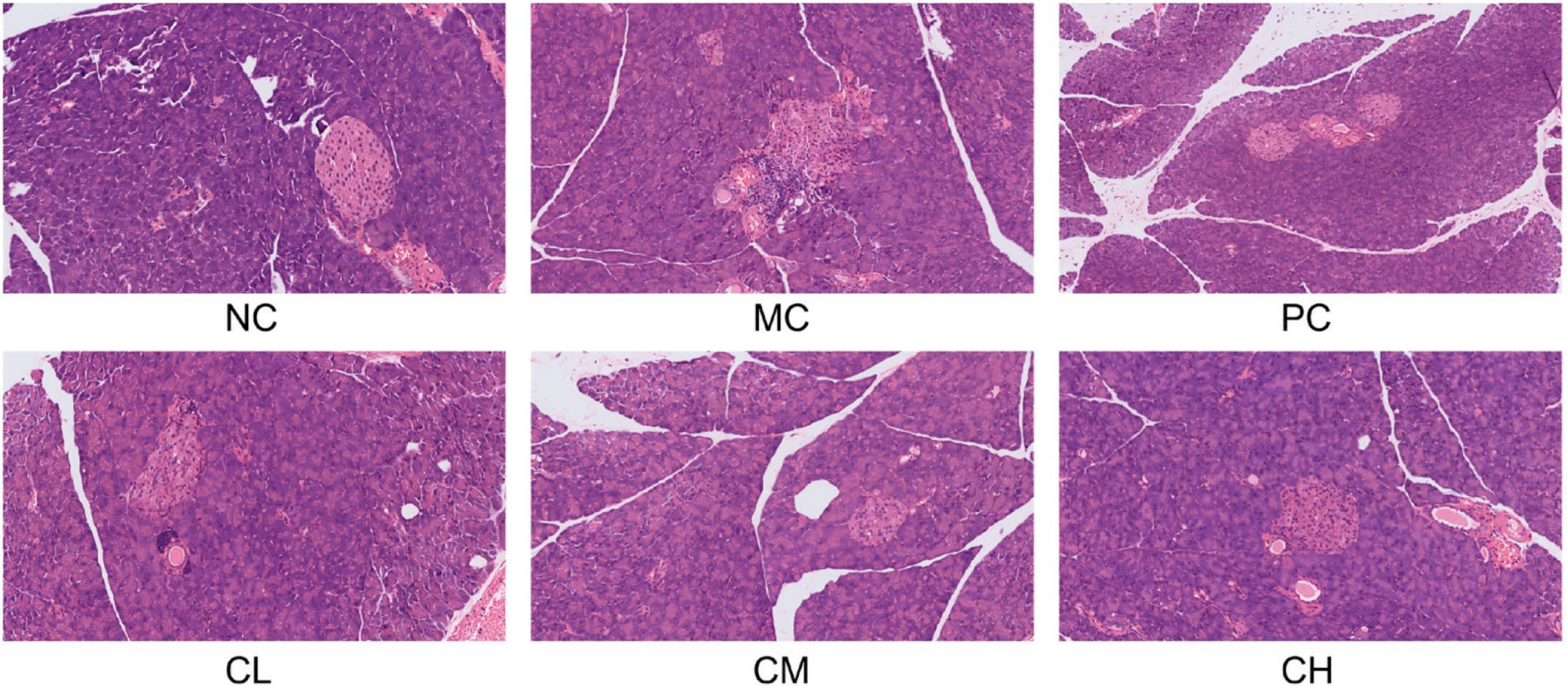
HE Staining of Pancreas in Mice from Different Groups (Magnification: ×100; Scale Bar: 100 μm).

As observed in Figure 6, the pancreatic tissue structure of the NC group was intact, with regularly shaped and evenly distributed islet cells, clear islet boundaries, and orderly arranged acinar cells, and no apparent pathological damage was evident. In contrast, the pancreatic tissue of the MC group exhibited significant pathological changes, including disrupted islet structure, shrunken and irregular cell morphology, widened islet interstitium accompanied by inflammatory cell infiltration, and disorganized acinar cell arrangement, indicating pronounced pancreatic injury and functional disturbance under diabetic conditions. After intervention in the PC group, the pancreatic pathological damage was ameliorated: the islet structure tended toward integrity, islet cell numbers partially recovered, the cell morphology was more regular than that in the MC group, and interstitial inflammatory infiltration was reduced.

The CL, CM, and CH groups all ameliorated pancreatic pathological damage in diabetic mice to varying degrees. In the CL group, the islet structure began to recover, and islet cell numbers showed an increase. In the CM group, the definition of islet boundaries was improved and the cell morphology was further normalized. In the CH group, pancreatic tissue morphology most closely approximated that of the NC group, with intact islet structure, markedly recovered islet cell numbers, regular cell morphology, essentially resolved interstitial inflammatory reactions, and a tendency toward orderly acinar cell arrangement (Peng et al. 2025). These findings indicate that CCP1A can attenuate pancreatic pathological injury by preserving pancreatic tissue morphology and maintaining islet cell integrity, and the effects exhibited a certain dose‐dependency.

#### 4.3.8 Liver Injury Markers Analysis

ALT and AST are core indicators of liver function status and are commonly used to assess the degree of hepatocyte injury. As functional enzymes within hepatocytes, they are released into the bloodstream in large quantities when diabetes induces hepatocyte damage, and elevated activities of these enzymes typically suggest the presence of liver injury.

**FIGURE 7:**
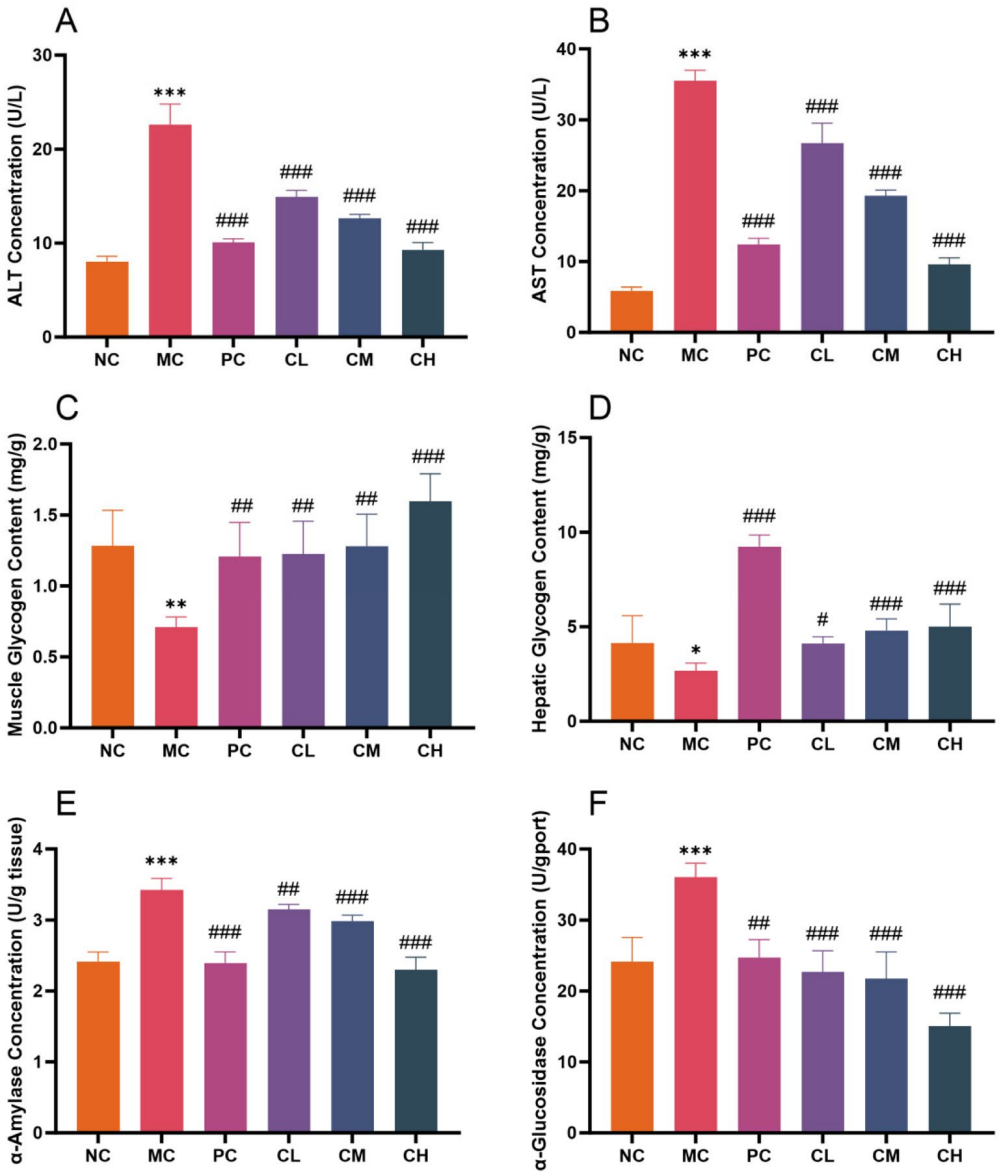
Serum ALT (A) and AST (B) levels, effects of CCP1A on muscle glycogen (C) and hepatic glycogen (D) contents, and α‐amylase (E) and α‐glucosidase (F) contents in the small intestines of mice from different groups. All data are shown as mean ± SD (*n* = 10). *p < 0.05, **p < 0.01, ***p < 0.001 versus the NC group, and ^#^ *p <* 0.05, ^##^ *p <* 0.01, ^###^ *p <* 0.001 versus the MC group.

As shown in Figure 7A,B, serum ALT and AST concentrations in the MC group were significantly higher than those in the NC group (*p <* 0.001), confirming that the diabetic state led to significant hepatocyte injury. After CCP1A intervention, the liver injury markers were improved in all treatment groups. Specifically, the ALT and AST values in the PC group were significantly lower than those in the MC group (*p <* 0.001). The CCP1A dose groups also exhibited a clear dose‐dependent ameliorative effect: the CL and CM groups showed a decreasing trend in these indicators, while the ALT and AST concentrations in the CH group were significantly lower than those in the MC group (*p <* 0.001) and were not significantly different from those in the PC group (*p* > 0.05). These results indicate that CCP1A, particularly at high dose, can significantly alleviate diabetes‐induced liver injury.

#### 4.3.9 Glycogen Content Analysis

Glycogen is the primary storage form of glucose in the body, and its synthesis and degradation are key steps in maintaining glucose homeostasis. Under diabetic conditions, insulin resistance leads to impaired hepatic glycogen synthesis and reduced muscle glycogen reserves, which is one of the important pathological mechanisms underlying persistent hyperglycemia (Song et al. 2026). As shown in Figure 7C,D, regarding muscle glycogen, the muscle glycogen content in the MC group was significantly decreased compared with the NC group (*p <* 0.01), indicating that diabetes impaired glucose uptake and glycogen storage function in skeletal muscle. Compared with the MC group, the muscle glycogen content in the PC, CL, and CM groups was significantly increased (*p <* 0.01), and it was highly significantly increased in the CH group (*p <* 0.001), with an effect comparable to that of the PC group. These results demonstrate that CCP1A can ameliorate the deficient hepatic and muscle glycogen reserves in diabetic mice in a dose‐dependent manner. The promoting effect on hepatic glycogen synthesis was evident even at the low dose (*p <* 0.05) and was enhanced with increasing dose, while the restorative effect on muscle glycogen reached a significant level at the medium dose (*p <* 0.01) and became more pronounced at the high dose.

Regarding hepatic glycogen, the hepatic glycogen content in the MC group was decreased compared with the NC group (*p <* 0.05), suggesting that the hepatic glycogen synthesis capacity was impaired under the diabetic state. Compared with the MC group, the hepatic glycogen content in the PC group was highly significantly increased (*p <* 0.001), in the CL group was significantly increased (*p <* 0.05), in the CM group was significantly increased (*p <* 0.01), and in the CH group was also highly significantly increased (*p <* 0.001).

#### 4.3.10 Digestive Enzyme Activity Analysis

α‐Amylase and α‐glucosidase are key enzymes that regulate carbohydrate digestion and absorption. α‐Amylase catalyzes the initial hydrolysis of starch into oligosaccharides, and its activity is defined as the amount of enzyme that produces 1 mg of reducing sugar per gram of tissue per minute. α‐Glucosidase, located in the brush border of the small intestine, is responsible for the final hydrolysis of oligosaccharides and disaccharides into glucose and represents the rate‐limiting step in intestinal glucose absorption; its activity is defined as the amount of enzyme that generates 1 μmol of product per gram of tissue protein per minute at 37°C. These two enzymes function cooperatively to determine postprandial blood glucose levels, and simultaneous measurement of their activities allows a comprehensive evaluation of the interventional effects of CCP1A on the entire process of carbohydrate digestion (Xiu et al. 2025).

As shown in Figure 7E,F, the α‐amylase activity in the small intestine of mice in the MC group was significantly higher than that in the NC group (*p <* 0.001), suggesting a compensatory enhancement of starch digestion capacity in the diabetic state, which may exacerbate the postprandial glucose load. Compared with the MC group, the enzyme activity in the PC group was highly significantly reduced (*p <* 0.001), returning to a near‐normal level. Following CCP1A intervention, the CL group showed a significant decrease compared with the MC group (*p <* 0.01), and both the CM and CH groups exhibited highly significant decreases (*p <* 0.001), with the inhibitory effect increasing in a dose‐dependent manner; the effect in the CH group was comparable to that in the PC group, indicating that CCP1A can effectively inhibit small intestinal α‐amylase activity and reduce the initial breakdown of starchy substrates.

The α‐glucosidase activity in the small intestine of mice in the MC group was highly significantly elevated compared with the NC group (*p <* 0.001), confirming the overactivation of the intestinal glucose production pathway in the diabetic state. After intervention, the PC group showed a significant reduction compared with the MC group (*p <* 0.01). All CCP1A dose groups exhibited highly significant inhibitory effects, with the differences in the CL, CM, and CH groups reaching a highly significant level compared with the MC group (*p <* 0.001), and a clear dose‐dependent relationship was observed. The CH group exhibited the lowest enzyme activity, with an inhibitory potency even superior to that of the PC group. These results demonstrate that CCP1A exerts a potent inhibitory effect on α‐glucosidase and can effectively block the final conversion of oligosaccharides to glucose.

#### 4.3.11 Oxidative Stress Markers and Antioxidant Capacity Analysis

**FIGURE 8:**
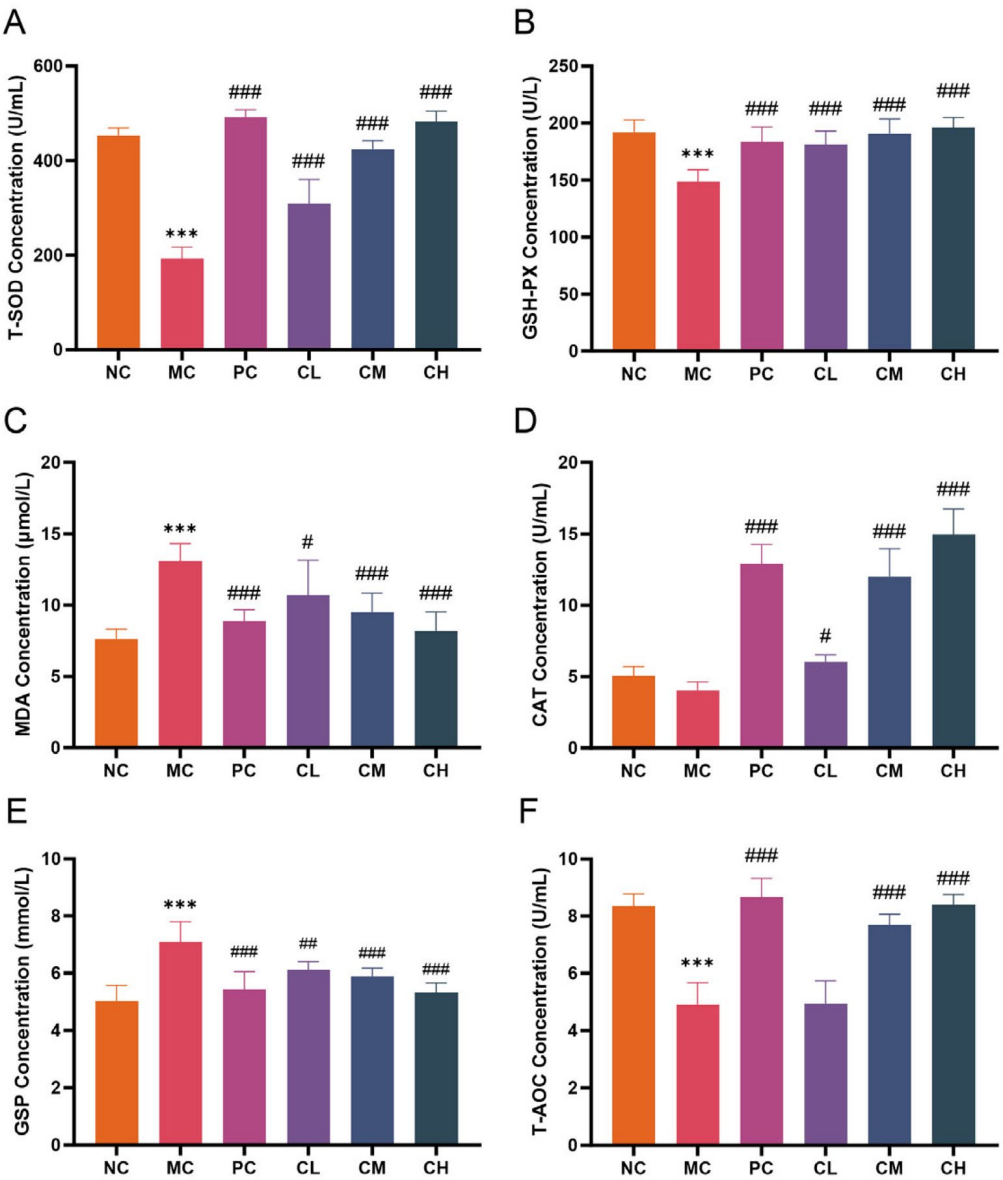
Effects of CCP1A on serum oxidative stress and glycation indicators in mice from different groups. (A) T‐SOD activity; (B) GSH‐Px activity; (C) MDA content; (D) CAT activity; (E) GSP content; (F) T‐AOC level. All data are shown as mean ± SD (*n* = 10). ***p < 0.001 versus the NC group, and ^#^ *p <* 0.05, ^##^ *p <* 0.01, ^###^ *p <* 0.001 versus the MC group.

T‐SOD is the primary defense line for scavenging oxygen free radicals in the body, maintaining the oxidation‐antioxidation balance by dismutating superoxide anions, and its activity directly reflects the body's antioxidant capacity. In Figure 8A, serum T‐SOD activity in the MC group was significantly decreased compared with the NC group (*p <* 0.001). Compared with the MC group, T‐SOD activity in the PC group was highly significantly increased (*p <* 0.001), and the CL, CM, and CH groups also exhibited significant upregulation (*p <* 0.001 versus the MC group), with an increasing trend along with dose, suggesting that CCP1A can effectively enhance the body's ability to scavenge oxygen free radicals by increasing SOD activity (Chang et al. 2025).

GSH‐Px is an important intracellular antioxidant enzyme that can eliminate free radicals and lipid peroxides, block oxidative chain reactions, and protect cell membrane structural and functional integrity (Mei et al. 2025). In Figure 8B, serum GSH‐Px activity in the MC group was significantly lower than that in the NC group (*p <* 0.001), indicating that the peroxide scavenging capacity was severely impaired in the diabetic state. Compared with the MC group, GSH‐Px activity in the PC group was highly significantly elevated (*p <* 0.001), and the CL, CM, and CH groups also displayed significant increases (*p <* 0.001), with the CH group showing a particularly pronounced recovery, suggesting that CCP1A can effectively activate GSH‐Px‐mediated antioxidant defense.

As the end product of lipid peroxidation, the serum level of MDA directly reflects the degree of cell membrane oxidative damage and the body's oxidative stress status. Figure 8C shows that serum MDA content in the MC group was significantly higher than that in the NC group (*p <* 0.001), confirming that T2D induced severe lipid peroxidation damage. After intervention, MDA levels in the PC group were highly significantly decreased compared with the MC group (*p <* 0.001), and the CM and CH groups also exhibited significant downregulation (*p <* 0.001 vs. the MC group), indicating that CCP1A can effectively suppress lipid peroxidation and alleviate oxidative damage under diabetic conditions.

CAT, as a key component of the antioxidant enzyme system, can specifically catalyze the decomposition of hydrogen peroxide into water and oxygen, preventing excessive generation of hydroxyl radicals. Figure 8D shows that serum CAT activity in the MC group was significantly lower than that in the NC group (*p <* 0.001), demonstrating that the hydrogen peroxide scavenging pathway was obstructed in the diabetic state. Compared with the MC group, CAT activity in the PC group was highly significantly elevated (*p <* 0.001); the CL group showed a significant increase (*p <* 0.05), and the CM and CH groups exhibited highly significant increases (*p <* 0.001). These results clearly demonstrate a classic dose‐dependent enhancement of CAT activity by CCP1A, with the medium‐ and high‐dose effects being significantly superior to the low‐dose effect (Liu, Zhang, and Zhu 2025).

GSP reflects the average blood glucose level over the preceding 2–3 weeks and is a sensitive indicator for evaluating short‐term glycemic control. Figure 8E shows that serum GSP content in the MC group was significantly higher than that in the NC group (*p <* 0.001), suggesting persistently high blood glucose in the recent period. Compared with the MC group, GSP content in the PC group was highly significantly decreased (*p <* 0.001); the CL group showed no significant reduction (*p* > 0.05), while both the CM and CH groups exhibited significant reductions (*p <* 0.001), with the CH group demonstrating the most pronounced effect. These results indicate that the improvement of short‐term glycated protein levels by CCP1A is dose‐dependent, with significant effects only observed at medium and high doses (Chen, Ge, et al. 2025).

T‐AOC is a core indicator that comprehensively evaluates the total levels of enzymatic and non‐enzymatic antioxidants in the body, reflecting the overall oxidative defense function (Shao et al. 2025). In Figure 8F, serum T‐AOC in the MC group was significantly decreased compared with the NC group (*p <* 0.001), confirming that the diabetic mice were in a state of systemic oxidative stress. Compared with the MC group, T‐AOC in the PC group was highly significantly elevated (*p <* 0.001); the CL group showed an increasing trend but without statistical significance (*p* > 0.05); the CM group exhibited a significant increase (*p <* 0.001); and the CH group showed a highly significant increase (*p <* 0.001), with the effect approaching that of the PC group, indicating that a certain dosage of CCP1A is required to effectively enhance the body's comprehensive antioxidant capacity.

Overall, CCP1A exerted a significant ameliorative effect on oxidative damage in T2D mice: the regulation of T‐SOD, GSH‐Px, and MDA showed significant effects at all doses (*p <* 0.05), whereas the improvements in CAT, T‐AOC, and GSP displayed a clear dose dependency. The low dose of CCP1A had limited effects, while the medium and high doses (especially the high dose) could reach or approach the interventional level of the positive drug.

#### 4.3.12 mRNA Expression Analysis

**FIGURE 9:**
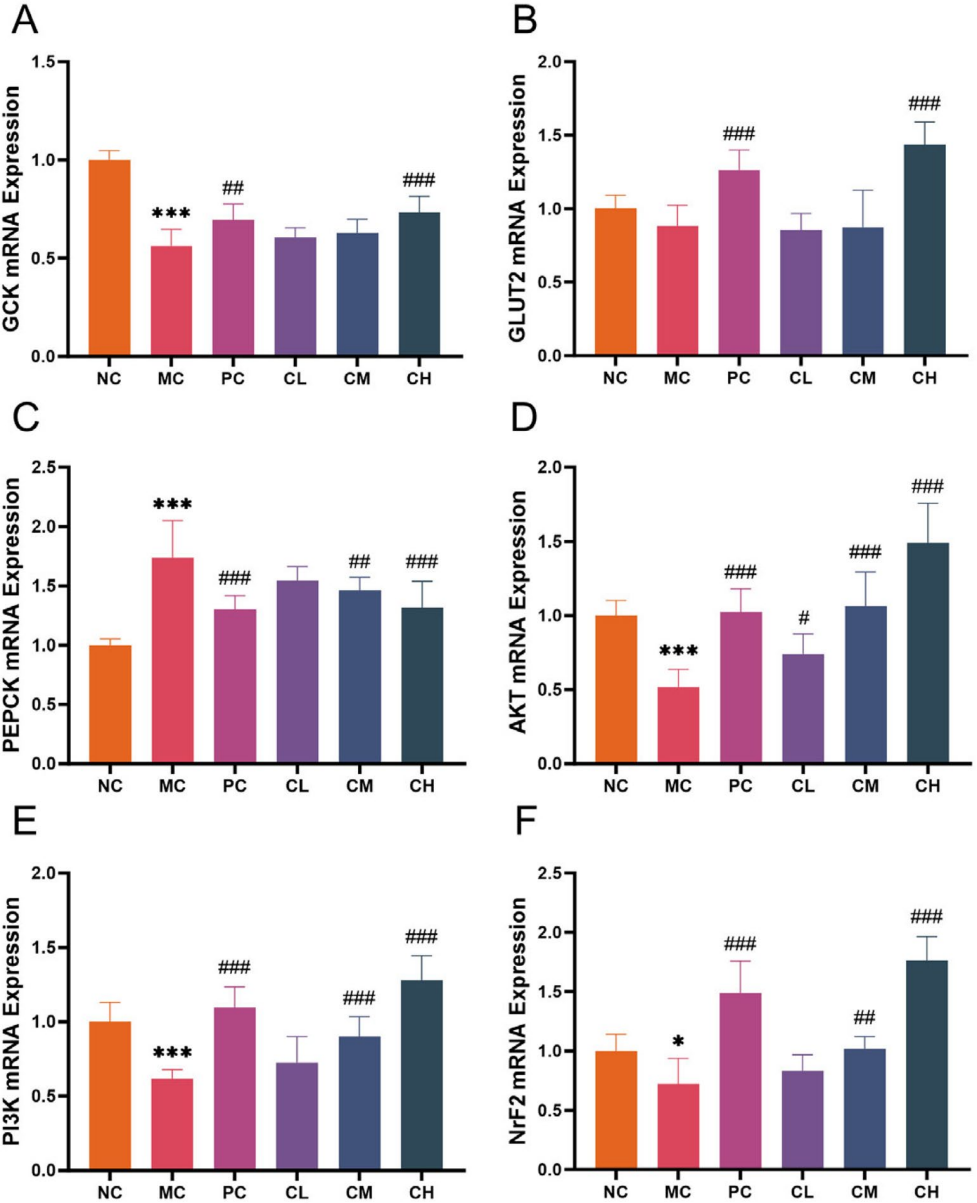
Effect of CCP1A on mRNA expression of genes related to glucose metabolism, insulin signaling, and antioxidant defense in the liver of diabetic mice. (A) GCK; (B) GLUT2; (C) PEPCK; (D) AKT; (E) PI3K; (F) Nrf2. All data are shown as mean ± SD (*n* = 3). *p < 0.05, ***p < 0.001 versus the NC group, and ^#^ *p <* 0.05, ^##^ *p <* 0.01, ^###^ *p <* 0.001 versus the MC group.

GCK, GLUT2, and PEPCK are core molecular markers in the regulation of hepatic glucose metabolism. GCK and GLUT2 synergistically promote glucose uptake and phosphorylation in hepatocytes, whereas PEPCK is the rate‐limiting enzyme of the gluconeogenic pathway, and its expression level directly reflects hepatic gluconeogenic activity. Figure 9A–C shows the relative mRNA expression of GCK, GLUT2, and PEPCK in the livers of Kunming mice from different groups, respectively.

Regarding GCK mRNA expression, compared with the NC group, the GCK mRNA expression level in the livers of mice in the MC group was highly significantly decreased (*p <* 0.001), indicating that hepatic glucose sensing and processing capacity were severely impaired under the diabetic state. Compared with the MC group, GCK expression in the PC group was significantly upregulated (*p <* 0.01), and the CCP1A dose groups also showed varying degrees of upregulation. Among them, the low‐ and medium‐dose CCP1A groups exhibited an upward trend but without statistical significance (*p* > 0.05), whereas GCK mRNA expression in the CH group was highly significantly increased (*p <* 0.001). Notably, a clear positive correlation was observed between the GCK expression level in the CCP1A dose groups and the administered dose, with the CH group essentially restoring expression to physiological levels, indicating that its promoting effect on hepatic glucokinase transcription is significantly dose‐dependent.

In terms of GLUT2 mRNA expression, compared with the NC group, GLUT2 expression in the livers of the MC group was slightly downregulated, but with no statistical significance (*p* > 0.05). Compared with the MC group, GLUT2 expression in the PC group was highly significantly upregulated (*p <* 0.001). The low‐ and medium‐dose CCP1A groups showed an upward trend without statistical significance (*p* > 0.05), whereas GLUT2 mRNA expression in the CH group was highly significantly increased (*p <* 0.001). These results suggest that a higher dose of CCP1A is required to exert a definitive regulatory effect on GLUT2 (Ma et al. 2025).

As for PEPCK mRNA expression, compared with the NC group, PEPCK expression in the MC group was highly significantly elevated (*p <* 0.001), confirming the excessive activation of the hepatic gluconeogenic pathway in diabetic mice. Compared with the MC group, PEPCK mRNA expression in the PC group was highly significantly decreased (*p <* 0.001), and all CCP1A dose groups also exhibited an inhibitory trend. The CL group showed no statistical significance (*p* > 0.05), the CM group displayed a significant decrease (*p <* 0.01), and the CH group showed a highly significant decrease (*p <* 0.001), with a marked reduction and an effect comparable to that of the PC group. These results demonstrate that CCP1A can dose‐dependently inhibit the expression of the key gluconeogenic enzyme and effectively block aberrant hepatic glucose output.

The PI3K/AKT signaling pathway is the core pathway through which insulin exerts its hypoglycemic effect (Zhang, Cao, et al. 2025). As shown in Figure 9D, regarding AKT mRNA expression, compared with the NC group, AKT expression in the MC group was highly significantly downregulated (*p <* 0.001). Compared with the MC group, AKT mRNA expression in the PC group was highly significantly upregulated (*p <* 0.001); the CCP1A low‐dose group exhibited a significant increase (*p <* 0.05), and both the CM and CH groups showed highly significant increases (*p <* 0.001).

As shown in Figure 9E, regarding PI3K mRNA expression, compared with the NC group, the PI3K expression level in the MC group was extremely significantly decreased (*p <* 0.001), indicating impaired insulin signaling pathway transduction. Compared with the MC group, the PI3K mRNA expression level in the PC group was extremely significantly restored (*p <* 0.001). The CCP1A low‐dose group showed an increasing trend but without statistical significance (*p* > 0.05), while both the CM and CH groups exhibited extremely significantly elevated expression levels (*p <* 0.001). These results indicate that CCP1A is associated with upregulated mRNA expression of PI3K and AKT. The coordinated changes in PI3K and AKT mRNA suggest a possible modulatory effect of CCP1A on insulin signaling‐related gene expression, although direct evidence of pathway activation at the protein or phosphorylation level is not yet available. Therefore, while CCP1A may influence hepatic glucose metabolism partly through transcriptional regulation of insulin signaling‐associated genes, conclusions regarding functional restoration of the insulin signaling pathway require further validation (Luo et al. 2025).

Nrf2 is a core transcription factor in cellular antioxidant responses, regulating the expression of antioxidant enzyme genes (Zhang, Liu, et al. 2025). As shown in Figure 9F, compared with the NC group, Nrf2 mRNA expression in the MC group was significantly decreased (*p <* 0.05), indicating impaired transcriptional regulation of the antioxidant defense system in diabetic mice. Compared with the MC group, Nrf2 mRNA expression in the PC group was extremely significantly upregulated (*p <* 0.001). The CCP1A low‐dose group showed an increasing trend but without statistical significance (*p* > 0.05), while the CM group showed a significant increase (*p <* 0.01) and the CH group showed an extremely significant increase (*p <* 0.001). These results suggest that the antioxidant effect of CCP1A may involve Nrf2‐related gene expression, but definitive mechanistic conclusions require protein‐level and functional validation. This alleviation of oxidative stress not only directly protects tissues from ROS damage but also indirectly contributes to improved glycemic control by ameliorating the redox microenvironment of pancreatic β‐cell function and insulin signaling, as further supported by the significant decrease in GSP levels.

CCP1A significantly regulates the mRNA expression of genes involved in hepatic glucose metabolism, the insulin signaling pathway, and the antioxidant defense system. Among these, the regulation of GCK and PEPCK mRNA is significant at all doses, whereas the upregulation of GLUT2 mRNA requires a high dose to become apparent. The significant activation of PI3K, AKT, and Nrf2 mRNA is mainly observed at medium and high doses, collectively demonstrating a favorable dose‐effect relationship. These mRNA‐level changes suggest potential modulation of insulin signaling and antioxidant responses, but functional validation at the protein and pathway levels is needed to confirm these effects.

## 5 Discussion

Compared with existing studies, this study obtained a high purity polysaccharide CCP1A with a relatively clear structure through extraction and purification, and performed systematic structural characterization. The purity, protein content, and uronic acid content of CCP1A were 89.41% ± 0.95%, 1.27% ± 0.11%, and 23.46% ± 0.21%, respectively. CCP1A is a heteropolysaccharide with a moderate molecular weight. Its weight average molecular weight (Mw) is 8.161 kDa, the polydispersity index is 1.317, indicating a moderately broad molecular weight distribution. The monosaccharide composition is dominated by glucose and galacturonic acid, with small amounts of rhamnose, arabinose, galactose, etc. CCP1A simultaneously possesses a high proportion of linear homogalacturonan (HG) domains and a highly branched rhamnogalacturonan I (RG I) domain. FT IR spectroscopy confirmed typical polysaccharide absorption peaks, with sugar units existing in pyranose rings and containing both α and β glycosidic linkages. ^1^H NMR spectra further revealed the complexity of its glycosidic linkages. The Congo red test indicated that CCP1A forms a stable helical conformation in aqueous solution. Transmission electron microscopy revealed a dendritic, irregularly dispersed aggregate morphology with curly chain segments, consistent with the helical conformation characteristics. It should also be noted that the fine structural details of CCP1A, such as the specific glycosidic linkage patterns and the main chain/branch architecture, have not been fully resolved. Therefore, any structure–activity relationship inferred from the current data remains preliminary and requires validation through more advanced structural analyses (e.g., methylation analysis, 2D NMR).

Unlike most studies that focus on crude cinnamon polysaccharides or only perform in vitro activity assays, this paper, for the first time, conducted a systematic in vivo evaluation ranging from whole‐animal phenotypes, organ pathology to hepatic gene expression, and elucidated the in vivo inhibitory effects of CCP1A on intestinal digestive enzymes, its in vivo antioxidant effects, as well as its amelioration of typical diabetic symptoms such as body weight, food intake, and water intake.

CCP1A significantly lowered fasting blood glucose, improved glucose tolerance, and alleviated polydipsia, polyphagia, weight loss, dyslipidemia, as well as pathological damage to the pancreas in type 2 diabetic mice. On one hand, CCP1A inhibits α‐amylase and α‐glucosidase in the intestine, delaying glucose absorption. On the other hand, in the liver, it regulates the expression of glucose metabolism‐related genes, specifically by increasing the mRNA levels of GCK and GLUT2 and decreasing the mRNA level of PEPCK, thereby promoting hepatic glycogen synthesis and inhibiting hepatic gluconeogenesis. Furthermore, CCP1A tended to upregulate Nrf2 mRNA expression, enhance the activities of T‐SOD, GSH‐Px, and CAT, improve overall antioxidant capacity, reduce lipid peroxidation and protein glycation, and thereby ameliorate systemic oxidative stress in diabetic mice. However, these observations are derived solely from mRNA expression data (including PI3K/AKT‐ and Nrf2‐related genes) and serum biochemical indicators. Direct evidence supporting the restoration of insulin signaling or full activation of the PI3K/AKT and Nrf2‐mediated antioxidant pathways—such as protein expression levels, phosphorylation status (e.g., p‐AKT, p‐PI3K), or pathway‐specific functional assays—is currently lacking. Therefore, the current findings indicate associations only at the gene expression level and are insufficient to confirm definitive activation of the relevant signaling pathways. Future in‐depth mechanistic analyses, including molecular docking, validation of key target binding, and investigation of intermolecular interaction modes, are warranted. It should be objectively noted that the observed improvements in oxidative stress and the alleviation of pancreatic and hepatic pathological damage in this study may result either from direct effects of CCP1A or from secondary protective effects due to improved blood glucose, and these two factors are difficult to completely distinguish. These multi‐target regulatory characteristics align with the comprehensive advantages of natural polysaccharides and constitute a complete regulatory chain of CCP1A, from reducing glucose sources and enhancing tissue glucose utilization to repairing oxidative damage, thus providing a theoretical basis for developing it as a hypoglycemic functional food.

Metformin primarily improves insulin resistance and inhibits hepatic gluconeogenesis, while acarbose specifically inhibits intestinal α‐amylase and α‐glucosidase. Their combination simultaneously covers the two core links: inhibition of intestinal glucose absorption and regulation of hepatic glucose metabolism. Therefore, using metformin combined with acarbose as the positive control in this study can comprehensively reflect the multi‐target, multi‐pathway intervention characteristics of CCP1A. However, this study did not include an acarbose‐alone control group, making it difficult to directly compare the intestinal glycosidase inhibitory effect of CCP1A with that of acarbose, thus presenting a limitation in directly comparing enzyme inhibitory activities.

In addition, several limitations of this study should be acknowledged. The pharmacokinetic characteristics of CCP1A in vivo (e.g., absorption, distribution, and metabolism) and its direct molecular targets remain unclear. Key aspects such as oral bioavailability, in vivo metabolic processes, tissue distribution, and targeted delivery efficiency of this polysaccharide have not been sufficiently addressed. Furthermore, structural modifications of cinnamon polysaccharides—such as sulfation, acetylation, and carboxymethylation—as well as modification strategies to enhance efficacy, warrant further investigation. For instance, Chen et al. (2026) demonstrated that selenylation modification of a purified cinnamon polysaccharide greatly improved its anti‐melangenic activity. Future research should more comprehensively investigate the in vivo absorption and metabolism of CCP1A, explore whether it directly binds to and interacts with key targets such as the insulin receptor, and conduct long‐term safety evaluations.

## Author Contributions

**Yimiao Zhou:** conceptualization, writing – original draft. **Lin Yang:** investigation. **Tianjin Ma:** data curation, formal analysis. **Chen Ding:** methodology, investigation. **Xiao Liu:** formal analysis, data curation. **Liquan Zhou:** conceptualization, methodology, investigation, writing – original draft, data curation, formal analysis. **Zuowei Xiao:** writing – review and editing, funding acquisition, supervision.

## Funding

This research was funded by the Hunan Natural Science Foundation (Grant No. 2024JJ8163); the Natural Drug Resources and Function Development Fund Project (Grant No. 2022ZYYGN06); the project “Analysis and Development Research on the Current Status of Medicinal and Edible Homologous Industries in Hunan Province” (Grant No. 2025JCTJA60); and the Agricultural Technology System of Traditional Chinese Medicine in Guizhou Province (Grant No. GZZYCCYJSTX‐02).

## Ethics Statement

This study received approval from the Institutional Animal Ethics Committee of Hunan University of Chinese Medicine (protocol HNUCM21‐2506‐20; approval date: 20 June 2025).

## Consent

The authors have nothing to report.

## Conflicts of Interest

The authors declare no conflicts of interest.

## Data Availability

The data that support the findings of this study are available from the corresponding author upon reasonable request.
